# CFTR Modulator Therapy Enhances Peripheral Blood Monocyte Contributions to Immune Responses in People With Cystic Fibrosis

**DOI:** 10.3389/fphar.2020.01219

**Published:** 2020-08-13

**Authors:** Katherine B. Hisert, Timothy P. Birkland, Kelly Q. Schoenfelt, Matthew E. Long, Brenda Grogan, Suzanne Carter, W. Conrad Liles, Edward F. McKone, Lev Becker, Anne M. Manicone, Sina A. Gharib

**Affiliations:** ^1^ Division of Pulmonary, Critical Care, and Sleep Medicine, Department of Medicine, National Jewish Health, Denver, CO, United States; ^2^ Center for Lung Biology, Division of Pulmonary, Critical Care, and Sleep Medicine, Department of Medicine, University of Washington, Seattle, WA, United States; ^3^ Ben May Department for Cancer Research, University of Chicago, Chicago, IL, United States; ^4^ Department of Medicine, St. Vincent’s University Hospital, Dublin, Ireland

**Keywords:** cystic fibrosis, monocytes, ivacaftor, inflammation, transcriptome

## Abstract

**Background:**

CFTR modulators decrease some etiologies of CF airway inflammation; however, data indicate that non-resolving airway infection and inflammation persist in individuals with CF and chronic bacterial infections. Thus, identification of therapies that diminish airway inflammation without allowing unrestrained bacterial growth remains a critical research goal. Novel strategies for combatting deleterious airway inflammation in the CFTR modulator era require better understanding of cellular contributions to chronic CF airway disease, and how inflammatory cells change after initiation of CFTR modulator therapy. Peripheral blood monocytes, which traffic to the CF airway, can develop both pro-inflammatory and inflammation-resolving phenotypes, represent intriguing cellular targets for focused therapies. This therapeutic approach, however, requires a more detailed knowledge of CF monocyte cellular programming and phenotypes.

**Material and Methods:**

In order to characterize the inflammatory phenotype of CF monocytes, and how these cells change after initiation of CFTR modulator therapy, we studied adults (n=10) with CF, chronic airway infections, and the *CFTR-R117H* mutations before and 7 days after initiation of ivacaftor. Transcriptomes of freshly isolated blood monocytes were interrogated by RNA-sequencing (RNA-seq) followed by pathway-based analyses. Plasma concentrations of cytokines and chemokines were evaluated by multiplex ELISA.

**Results:**

RNAseq identified approximately 50 monocyte genes for which basal expression was significantly changed in all 10 subjects after 7 days of ivacaftor. Of these, the majority were increased in expression post ivacaftor, including many genes traditionally associated with enhanced inflammation and immune responses. Pathway analyses confirmed that transcriptional programs were overwhelmingly up-regulated in monocytes after 7 days of ivacaftor, including biological modules associated with immunity, cell cycle, oxidative phosphorylation, and the unfolded protein response. Ivacaftor increased plasma concentrations of CXCL2, a neutrophil chemokine secreted by monocytes and macrophages, and CCL2, a monocyte chemokine.

**Conclusions:**

Our results demonstrate that ivacaftor causes acute changes in blood monocyte transcriptional profiles and plasma chemokines, and suggest that increased monocyte inflammatory signals and changes in myeloid cell trafficking may contribute to changes in airway inflammation in people taking CFTR modulators. To our knowledge, this is the first report investigating the transcriptomic response of circulating blood monocytes in CF subjects treated with a CFTR modulator.

## Introduction 

Although chronic airways diseases are a leading cause of morbidity and mortality in the world, therapies that dampen airway inflammation without inducing broad systemic immunosuppression or exerting clinically significant side effects are still lacking for many of these diseases. In cystic fibrosis (CF), a disease of chronic airway infection and chronic inflammation, NSAIDs and steroids improve lung function and decrease symptoms (Cheng et al., 2013); however, adverse side effects limit their use (Mogayzel, Jr. et al., 2013; Cantin et al., 2015). Development of effective inflammation-dampening therapies for people with CF is further complicated by the fact that some components of the immune response are necessary for controlling airway infections, while others may only enhance tissue damage (Doring et al., 2014; Konstan et al., 2014), thus therapies ideally would target specific arms of the inflammatory response (Lin and Kazmierczak, 2017). However, the contributions of specific cell populations and inflammatory mediators to persistence of non-resolving inflammation remain incompletely understood in CF, like in many chronic lung diseases, largely due to a lack of animal models that recapitulate chronic airway inflammation,

Research in human subjects indicates that peripheral blood monocytes participate in inflammation in many chronic diseases by trafficking to sites of damage and infection where they develop into macrophages (Byers and Holtzman, 2011; Dewhurst et al., 2017; Kapellos et al., 2019). Because macrophages are long-lived cells that can initiate, modulate, and resolve inflammation (Johnston et al., 2012; Wynn and Vannella, 2016; Puttur et al., 2019), pharmacologic manipulation of monocytes and macrophages to shift these cells toward disease-resolving phenotypes has been suggested as a therapeutic strategy in a number of chronic inflammatory diseases including pulmonary fibrosis and cardiac disease (Cheng and Rong, 2018; Liu et al., 2019). In CF, neutrophils and macrophages make up the majority of cells in the airway lumen (Henig et al., 2001; Hisert et al., 2019), and many CF airway macrophages appear to be derived from blood monocytes (Wright et al., 2009; Garratt et al., 2012), thus medications that target peripheral blood monocytes could be an effective anti-inflammatory strategy in CF as well.

Cell-focused therapies require knowledge of both how the cell population participates in disease pathophysiology as well as how the disease state alters immune cell programming and function. Multiple studies have shown that CF monocytes and macrophages mount aberrant immune responses (Bruscia and Bonfield, 2016). Monocytes from people with CF demonstrate tolerance to LPS (del Fresno et al., 2008) and impaired adhesion and trafficking (Sorio et al., 2015) compared to cells from healthy donors, and some monocyte defects seen in CF can be induced in healthy donor cells by exposure to CF plasma (Zhang et al., 2019). In contrast, studies using cells from CFTR deficient animals (that have not developed chronic lung infections and inflammation) and human CF macrophages cultured *in vitro* indicate that lack of CFTR activity causes macrophages to mount overly robust inflammatory responses (Bruscia et al., 2009; Bonfield et al., 2012). Thus, monocytes that migrate to the CF airway and become macrophages could have abnormal immune responses for at least two distinct, but intertwined, reasons: a direct effect of lack of sufficient CFTR activity, and the secondary effects of exposure to plasma containing products of non-resolving infection and chronic disease. Understanding the *in vivo* phenotypes of CF monocytes and macrophages is an essential first step towards devising methods to manipulate the cellular programs of these key regulatory cells to help dampen or resolve inflammation.

As we enter the era of highly effective CFTR modulator therapy, drivers of inflammation in CF will change for patients receiving these medications. Inflammation resulting directly from a lack of CFTR activity will become less pronounced, if not eliminated, by CFTR modulators (Sorio et al., 2015; Barnaby et al., 2018; Rosen et al., 2018). However, studies indicate that chronic airway infection and inflammation, particularly in subjects with advanced lung disease, will continue to cause symptoms and progressive lung damage (Rowe et al., 2014; Heltshe et al., 2015; Hisert et al., 2017). Based on prior studies of CF monocytes and macrophages exposed *ex vivo* to CFTR modulators, restoration of CFTR activity could either enhance or dampen cellular responses (Barnaby et al., 2018; Zhang et al., 2018). In order to characterize the inflammatory phenotype of *in vivo* blood monocytes in people with CF, and to determine how cellular phenotypes change in response to CFTR modulator therapy, we have used unbiased “omics” methods to identify changes in freshly isolated peripheral blood monocytes following initiation of ivacaftor in people with susceptible *CFTR* mutations. Previously we identified changes in the monocyte plasma-membrane associated proteome in subjects with *CFTR-G551D* mutations that suggested that CFTR modulator therapy causes a decrease in monocyte IFN*γ* responses (Hisert et al., 2016), a hypothesis that we have since confirmed (Hisert et al., 2020). Here we describe how restoration of CFTR activity by ivacaftor acutely changes the peripheral blood monocyte transcriptome and plasma chemokines in a cohort of adults with CF and the *CFTR* mutation *R117H.*

## Materials and Methods

### Patient Cohort and Study Design

Ten patients (6 male and 4 female) from the Adult Cystic Fibrosis Clinic at St. Vincent’s University Hospital in Dublin, Ireland were enrolled in this study. Human subject recruitment was approved by the Research Ethics Committee, and all study participants provided written informed consent. Subjects were excluded if they had participated in the VX-770 (ivacaftor) Extended Access Program or had used ivacaftor within 6 months prior to the day 0 visit, or if they had required treatment with oral, inhaled or IV antibiotics within the 2 weeks prior to the day 0 visit. Subjects were,thus, considered at their clinical baseline at the day 0 visit. Subjects were allowed to continue other standard CF therapies. The day 0 visit for all subjects occurred during a two-week period in fall of 2016, and all subjects were started on ivacaftor treatment during the same week. At each study visit subjects provided sputum and blood specimens and underwent assessment of vital signs, weight, sweat chloride, and spirometry. Ivacaftor treatment was initiated following the day 0 study visit. Subjects were grouped into cohorts of three to four subjects on each day, and specimens from each cohort were processed in parallel on the same day.

### Processing of Blood and Isolation of Monocytes

**Figure 1:**
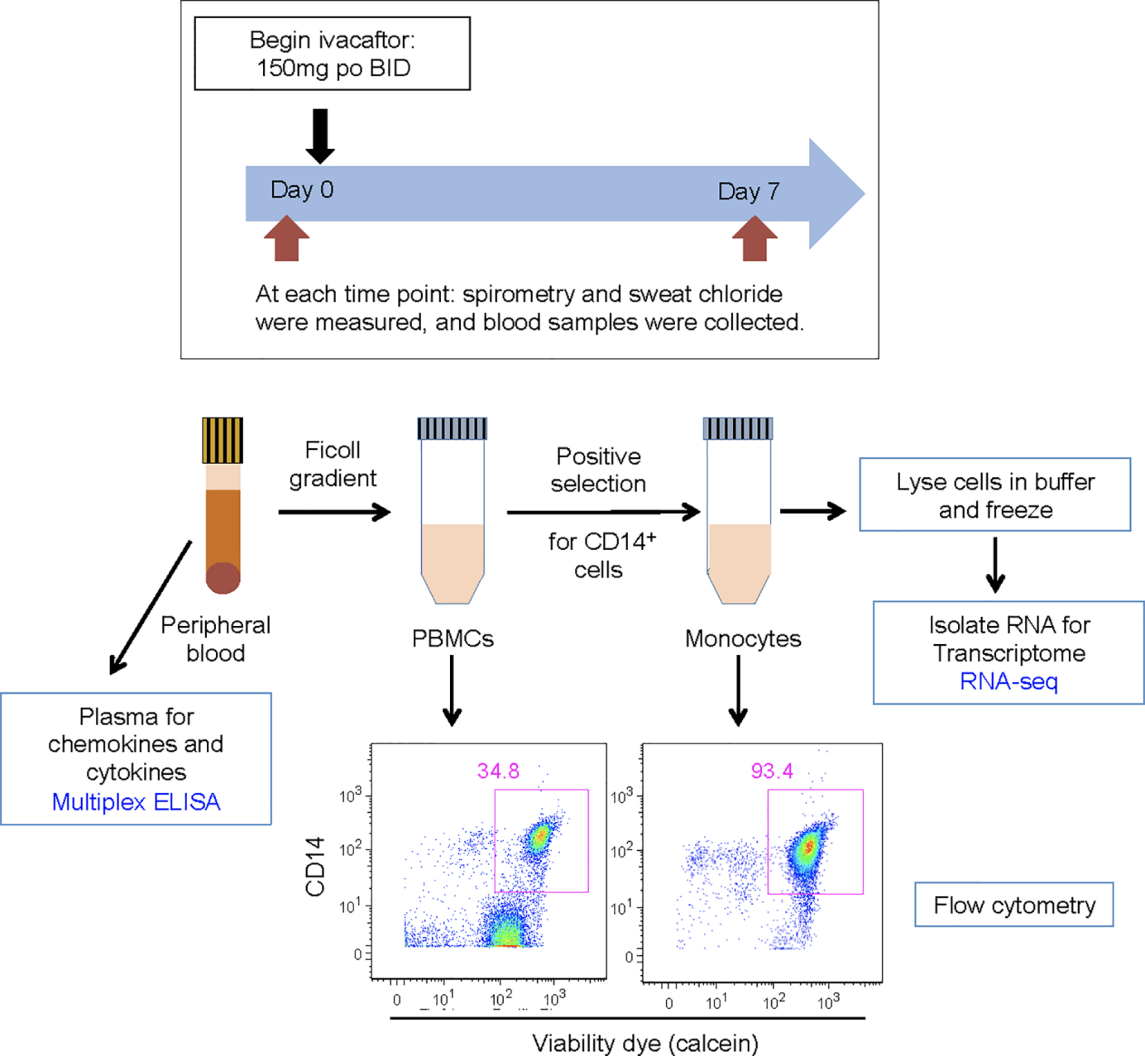
Schematic for study design. Subjects were evaluated on day 0 and day 7. Subjects initiated ivacaftor treatment following the day 0 study visit. During each visit, subjects underwent a physical examination and sweat chloride evaluation, performed spirometry, and had blood drawn. Blood samples, acquired into K-EDTA tubes, were brought to the laboratory on ice. One tube of blood was centrifuged to obtain plasma. The remaining blood was processed to isolate PBMCs and then monocytes. Aliquots of PBMCs and monocytes were removed for quantification of cells and flow cytometric analysis to determine abundance of leukocyte sub-populations and purity of isolated monocytes. Cells recovered from CD14 positive selection column were immediately lysed in RLT buffer (Qiagen) and frozen.

Whole blood was collected into K-EDTA tubes from subjects before (day 0) and seven days after initiation of ivacaftor (**Figure 1**). One aliquot was used for separation of plasma from blood cells. The remaining blood was separated using gradient centrifugation over Ficoll-paque (GE) to separate peripheral blood mononuclear cells (PBMCs) from neutrophils and red blood cells. The Miltenyi Monocyte Isolation Kit (a positive selection kit that uses magnetic beads conjugated to CD14 antibody) was used to isolate monocytes. Numbers of PBMCs and cells recovered by magnetic beads were quantified using a Neubauer hemocytometer, and values pre- and post-ivacaftor were compared using Student’s *t*-test. Efficacy of monocyte isolation was confirmed by assessing proportions of T cells (CD3+ cells), B-cells (CD19+ cells), and monocytes (CD14+ cells) before and after cells were subjected to the Monocyte Isolation Kit.

### Quantification of Plasma Cytokines and Chemokines

Plasma cytokines and chemokines were measured using a custom Human Magnetic Luminex pre-mixed multiplex assay (R&D Systems). Plasma levels of IL-8, IL-10, G-CSF, IL-6, CCL4, CXCL1, Il-1β, TNF, GM-CSF, and IL-12p40 were all below the limit of detection in most subjects at both time points. For those analytes that were detected, statistically significant differences between day 0 and day 7 were determined by using the Wilcoxon Signed-Rank test.

### RNA Isolation

RNA was isolated from human peripheral blood monocytes using Qiagen’s RNeasy Plus kit per manufacturer’s instructions. Briefly, freshly isolate monocytes were homogenized in Qiagen RLT buffer, snap frozen, and stored at −80°C until further processing *via* the Qiagen kit. Purified RNA from samples was eluted in Ambion’s RNA storage solution and stored frozen at −80°C until use.

### RNA Sequencing

RNA-seq was performed by the Genomics Core at the Benaroya Research Institute. For each sample, libraries were constructed from 100 ng of total RNA using the TruSeq Stranded mRNA kit (Illumina) with poly(A) selection. Libraries were pooled and quantified using a Qubit^®^ Fluorometer (Life Technologies). Single-read sequencing of pooled libraries was carried out on a HiSeq2500 sequencer (Illumina) with 58-base reads, using HiSeq v4 Cluster and SBS kits (Illumina) with a target depth of 10 million reads per sample. Basecalls were processed to FASTQs on BaseSpace (Illumina), and a base call quality trimming step was applied to remove low-confidence base calls from the ends of reads. The FASTQs were aligned to the human reference genome (GRCh38.91), using STAR v.2.4.2a (Dobin et al., 2013) and gene counts were generated using htseq-count (Anders et al., 2015). QC and metrics analysis were performed using the Picard family of tools (v1.134) (https://broadinstitute.github.io/picard/). All RNA-seq data meeting MINSEQE (Minimum Information About a Next-generation Sequencing Experiment) have been deposited at Gene Expression Omnibus repository (https://www.ncbi.nlm.nih.gov/geo/, GSE148076).

### RNA-Sequencing Data Analysis

To explore the overall changes in monocyte transcriptome in response to ivacaftor, we applied multidimensional scaling using Principal Components Analysis (PCA) to the entire RNA-seq profiles. Since each subject was assessed twice (day 0, day 7), we used a multilevel decomposition for repeated measures as implemented by the “mixOmics” package in R statistical environment (Rohart et al., 2017).

Differentially expressed genes between day 0 (untreated baseline) and day 7 (after ivacaftor therapy) were identified using paired statistical analysis for each subject (pre vs. post treatment) with “DESeq2” package in R (Love et al., 2014). Adjustment for multiple hypothesis testing was implemented using Benjamini-Hochberg’s false discovery rate (FDR) analysis, with an FDR < 0.05 designating significant differential gene expression. Two-dimensional hierarchical clustering of log_2_[Day 7/Day 0] gene expression ratios was performed using a Euclidian distance metric.

Gene product interaction network analysis was applied to the differentially expressed genes (FDR < 0.05) based on experimentally verified relationships derived from Ingenuity (Calvano et al., 2005) and STRING (v.11, https://string-db.org/) (Szklarczyk et al., 2019).

Pathway enrichment was performed using the Gene Set Enrichment Analysis (GSEA) program (Subramanian et al., 2005), where all unique transcripts were rank-ordered based on their DESeq2 statistic and over 7,000 gene sets derived from canonical pathways (Hallmark, KEGG, Reactome, Biocarta) and Gene Ontology (GO) annotations were assessed. An FDR < 0.05 was used to identify significant enrichment based on 1000 random gene set permutations. Enrichment Map (Isserlin et al., 2014), an application within the Cytoscape software platform (Cline et al., 2007), was used to create a network-based visualization of the GSEA results. To simplify the pathway enrichment network, only the most significantly enriched gene sets (FDR=0) were used as nodes. Edges were drawn between gene sets if at least 50% of their gene members overlapped. The emerging topology of the network allowed identification of aggregates of highly connected nodes that defined distinct biological modules. To compare our study with the GOAL cohort, we performed Gene Ontology enrichment analysis on differentially up and down-regulated genes between the subset of subjects that had clinical response to one month of ivacaftor therapy (“responders”) vs. those that did not (“non-responders”) using Webgestalt program (Liao et al., 2019). An enrichment FDR < 0.05 was used to designate significantly enriched processes.

## Results

### Cohort Characteristics

All subjects were heterozygous for the *R117H-CFTR* gene allele (5T Poly T tract), and seven subjects’ second mutation was *ΔF508;* none of the subjects had 2 ivacaftor-sensitive CFTR mutations (**Table 1**). Subjects were all adults with an age range of 25 – 52 years old (median, 40.5 years), baseline sweat ranged from 61 to 98 mM (median 79 mM), and body mass index (BMI) ranged from 18.5 to 32.5 (median 25.1). The cohort subjects demonstrated a wide range of baseline forced expiratory volume in one second (FEV_1_), from 35% to 109% (median, 72%); 3 subjects had a baseline FEV_1_ > 75% predicted, 5 subjects had a baseline FEV_1_ of 60% to 75% predicted, and 2 subjects had a baseline FEV_1_ < 50% predicted. None of the subjects had CF-related diabetes (CFRD); seven of the subjects were pancreatic sufficient, and three subjects were being evaluated for pancreatic insufficiency, but had recent normal fecal elastase values. Five subject had recent clinical sputum cultures positive for *Staphylococcus aureus (*± *Haemophilus*, *Pseudomonas* or *Acinetobacter* species), two subjects had recent *Pseudomonas aeruginosa* positive sputum cultures, three subjects had a history of *P. aeruginosa* positive sputum cultures, and one subject had not recently produced sputum. All subjects experienced a pronounced decrease in sweat chloride, and the cohort also experienced a statistically significant increase in FEV_1,_ within 48 h of ivacaftor treatment, and both changes were maintained at day 7 (Hisert et al., 2020).

**Table 1:** Subject Demographics.

Non-R117H CFTR mutation	7: ΔF508 2: M156R 1: 2622+1G→A
Age	40.5 years (25 – 52)
Gender	6 male; 4 female
BMI	25.1 (18.5 – 32.5)
Baseline sweat chloride (mM)	79 (61 – 98)
Baseline FEV_1_, % predicted	72% (35 – 109%)
Clinical laboratory sputum culture data	• 5 recent +*S. aureus (*± *Haemophilus*, *Pseudomonas* or *Acinetobacter* species) • 2 recent + *P. aeruginosa* • 3 prior + *P. aeruginosa* • 1 with no recent sputum production

Data presented as median (ranges) unless stated otherwise.

### Ivacaftor-Induced Changes in Plasma Chemokines Suggest Inflammatory Pathways Altered by CFTR Restoration Differ From Pathways Involved in CF Pulmonary Exacerbations

For this study, we chose to evaluate specimens at day 7 after subjects started ivacaftor because we hoped to identify changes that may be direct consequences of CFTR restoration by ivacaftor, rather than secondary effects of improved mucociliary clearance in the lung and decreased airway inflammation and bacterial burden. In our prior studies of subjects with *CFTR-G551D* mutations starting ivacaftor, GSEA analysis determined that proteomic changes that were significant at day 7 could be detected at day 2; however, most were not significant at day 2 (Hisert et al., 2016). Thus, although sweat chloride is changed by day 2 of treatment (Hisert et al., 2017), we deemed this timepoint likely too early to identify significant changes in plasma proteins and monocyte transcriptomes.

**Figure 2:**
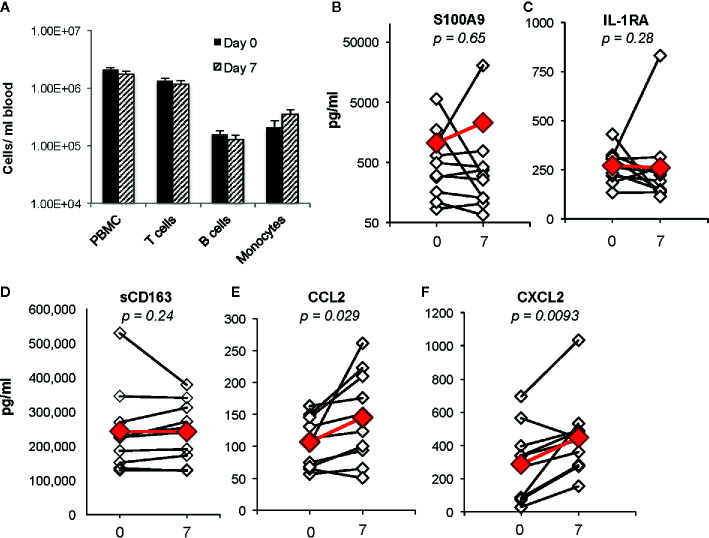
Changes in blood cells, proposed CF biomarkers, and chemokines following 1 week of ivacaftor therapy. **(A)** Total numbers of PBMCs/ml blood as determined by (i) quantitation of PBMCs using a hemocytometer and (ii) flow cytometric analysis of PBMCs (pre-selection for CD14+ cells) using antibodies for CD3 (T cells), CD19 (B cells), and CD14 (monocytes). **(B–F)** Plasma cytokine concentrations were determined in sample obtained before (day 0) and after (day 7) initiation of ivacaftor treatment using multiplex ELISA. Students’ *t*-test was used to compare data in panel **(A)**. Open simples with black lines are individual subjects. Red symbols indicate the mean value. Wilcoxon signed-rank test used to generate p-values for comparison of day 0 vs day 7 in **(B**–**F)**.

Identification of biomarkers that reflect systemic changes in inflammation in people with CF has been an area of intense interest, especially changes that predict CF pulmonary exacerbations and response to antibiotic therapy. Serum CRP level, calprotectin concentrations, and white blood cell counts decline following antibiotic treatment of exacerbations, and changes in some of these markers may predict response to therapy (Horsley et al., 2013; Sagel et al., 2015; Waters et al., 2015). We predicted that plasma biomarkers associated with decreases in bacterial burden in the airway would not be changed during the first week of ivacaftor therapy. In our prior cohort of subjects with *CFTR-G551D* mutations who started ivacaftor, we did not detect changes in plasma CRP levels or in numbers of PBMCs or monocytes, T cells or B cells in blood following 7 days of ivacaftor treatment (Hisert et al., 2016), consistent with our hypothesis that ivacaftor-induced changes in the airways during the first week of treatment likely had not yet translated to secondary effects in the systemic circulation. In the current cohort, we likewise observed that one week of ivacaftor treatment did not lead to detectable differences in numbers PBMCs and cells subsets (**Figure 2A**).

We then investigated whether ivacaftor changed levels of other plasma biomarkers that have been associated with inflammation in CF. Calprotectin, or S100A8/9, decreases in both sputum and serum following treatment of CF pulmonary exacerbations with antibiotics (Gray et al., 2010). In our study, we did not detect differences in levels of plasma S100A9 following initiation of ivacaftor (**Figure 2B**). We also found no changes in levels of plasma IL-1Ra (**Figure 2C**), a suppressor of inflammation that decreases in CF plasma during treatment of pulmonary exacerbations with intravenous antibiotics, and remains at lower levels during clinical stability as compared to during the exacerbation state (Sagel et al., 2015). CD163, a monocyte and macrophage surface protein that is shed when cells become activated (Davis and Zarev, 2005; Moller, 2012), has also been associated with CF disease state. Expression of CD163 mRNA is elevated in monocytes isolated from people with CF compared to cells from healthy controls, and was identified as part of a peripheral blood monocyte gene expression signature associated with successful treatment of CF pulmonary exacerbations (Saavedra et al., 2008; Nick et al., 2013). However in our study, there were no detectable changes in plasma levels of soluble CD163 (sCD163) at day 7 after initiation of ivacaftor (**Figure 2D**).

Although ivacaftor treatment did not change plasma levels of biomarkers associated with treatment of CF pulmonary exacerbations, we detected statistically significant increased plasma concentrations of two myeloid chemokines that are likely relevant to CF disease. CCL2 and CXCL2 were both increased at day 7 as compared to both pre-ivacaftor levels (**Figures 2E, F** **)**. CCL2, also known as monocyte chemoattractant protein 1, or MCP-1, is elevated in both sputum and plasma of people with CF as compared to healthy controls (Brennan et al., 2009; Rao et al., 2009). CXCL2, also known as macrophage inflammatory protein-2, or MIP-2, is a key chemokine for activation and recruitment of neutrophils to sites of inflammation, and is produced by monocytes, macrophages, epithelial cells, and other cells in response to injury (Huang et al., 2001; De Filippo et al., 2013). Free DNA content in human CF sputum correlates with both airway levels of CXCL2 and airflow obstruction, with increased airway free DNA and CXCL2 associating with lower FEV_1_, and similar findings were demonstrated in a CF mouse model (Marcos et al., 2015).

### Transcriptomic Analyses Reveal That Ivacaftor Treatment Activates Monocytes and Up-Regulates Inflammatory Pathways

**Figure 3:**
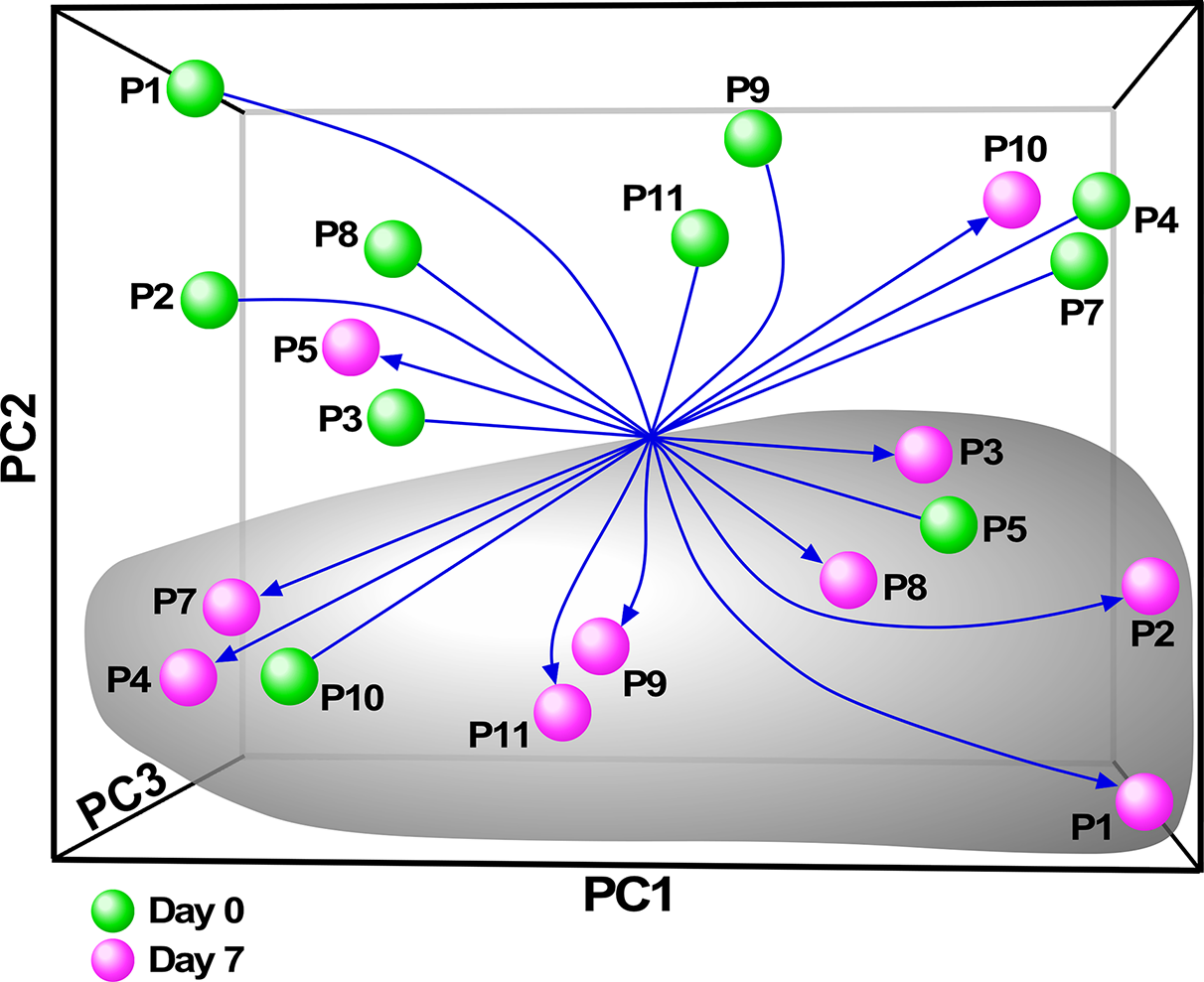
Principal components analysis (PCA) of monocyte transcriptome data. Multivariate decomposition PCA was applied to gene expression data from individual subjects pre-ivacaftor (day 0, green spheres) and post ivacaftor (day 7, magenta spheres) and plotted using the first three principal components (PC). Arrows are used to highlight the pre and post states. Despite variability across subjects, there is separation between day 0 and day 7 indicating that ivacaftor elicits a global transcriptional signal in monocytes.

Although there have been several published reports on gene expression profiling of PBMCs or whole blood from people with CF, we sought specifically to characterize the monocyte transcriptome, and determine how restoration of CFTR activity by initiation of ivacaftor acutely changes monocyte transcriptional signals. We thus performed RNA-seq on monocytes isolated from subjects before and 7 days after initiation of ivacaftor therapy. We initially performed exploratory analysis using Principal Components Analysis (PCA), a statistical method for reducing high dimensional data while retaining the drivers of expression variability, on the entire RNA-seq dataset. To capture the repeated measure structure of the study, we applied a multi-level implementation of PCA. We observed that most of the samples segregated based on pre- and post-ivacaftor time points (**Figure 3**), indicating that despite inter-individual variability, ivacaftor treatment led to global transcriptional changes in circulating monocytes.

**Figure 4:**
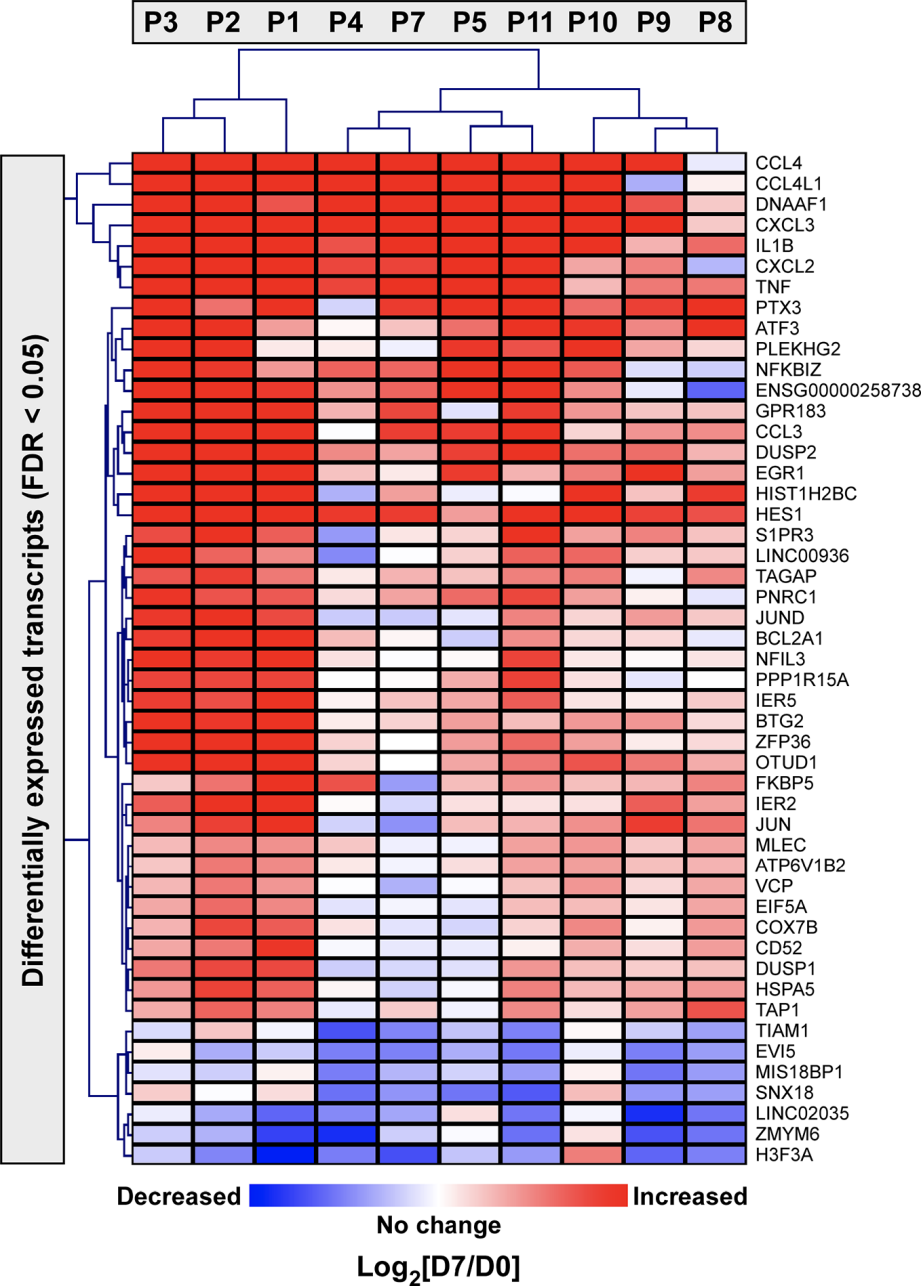
Two-dimensional hierarchical cluster analysis of statistically significantly differentially expressed monocyte genes in response to ivacaftor. The Heatmap depicts differentially expressed genes with FDR < 0.05 in rows and subjects in columns. The relative expression of each gene on day 7 vs. day 0 is shown with red indicating that the gene was increased in expression at day 7 compared to day 0, and blue indicating decreased expression at day 7 compared to day 0. Complete list with details is provided in **Supplementary Table S1**.

**Figure 5:**
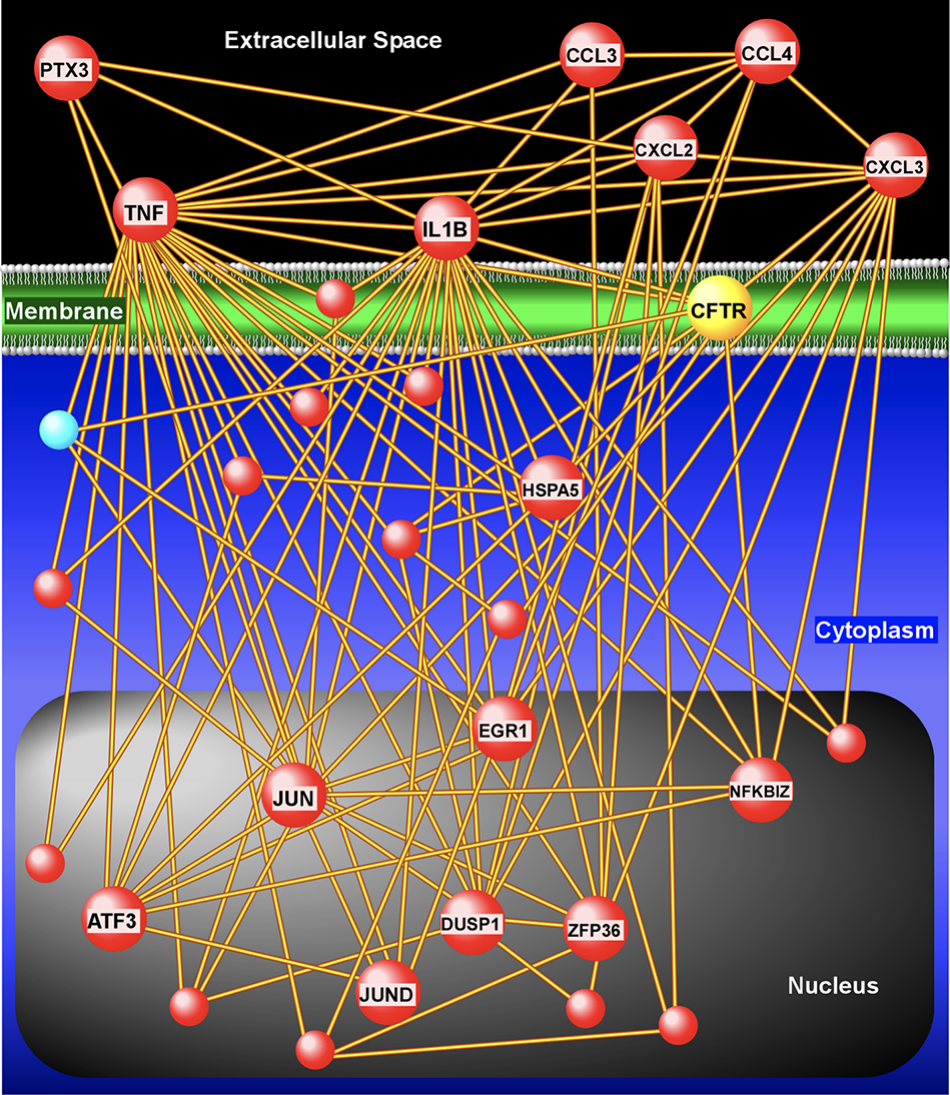
Gene product interaction network analysis of differentially expressed monocyte genes after 1 week of ivacaftor therapy. This “interactome” was constructed based on known relationships among the differentially expressed genes. To depict interaction of these nodes with the target of ivacaftor, we also added CFTR as a seed to the network. Note the presence of several highly connected nodes (or hubs), such as IL1B, TNF, CXCL3, CXCL2, that are up-regulated (red spheres) and are key modulators of immune signaling.

Next, we applied a subject-specific (paired) gene-based statistical method and identified 49 differentially expressed genes after adjustment for multiple comparisons (FDR < 0.05) with significant differences between pre-ivacaftor and day 7 post ivacaftor conditions **(** **Figure 4**, **Table S1**). Of these differentially expressed genes, 42 were up-regulated following ivacaftor treatment and 7 were down-regulated. The predominance of up-regulated genes was surprising because monocytes are key innate immune activators of inflammation, and ivacaftor has been shown to reduce markers of inflammation in people with CF (Hisert et al., 2017; Ronan et al., 2018), as well as cellular responses and markers of inflammation (Bratcher et al., 2016; Barnaby et al., 2018; Zhang et al., 2018). Also remarkable were the identities of the significantly up-regulated transcripts: many of these genes code for canonical pro-inflammatory factors, including cytokines (TNF, IL-1β) and chemokines (CCL4, CXCL2). To better elucidate the relationship among these differentially expressed genes, we performed gene product interaction network analysis. As depicted in **Figure 5** (with additional details in **Figure S1**), the resulting interactome revealed complex relationships among the network nodes and identified several densely connected “hubs,” such as TNF, IL1B, CXCL3, CXCL2, ZPF36, JUN, and DUSP1 that may be important drivers of the monocyte transcriptional response to ivacaftor treatment. Furthermore, by adding CFTR as a network node, we observed that several of these hubs (e.g., TNF, IL1B, CXCL3, JUN) also interact with the functional target of ivacaftor.

**Figure 6:**
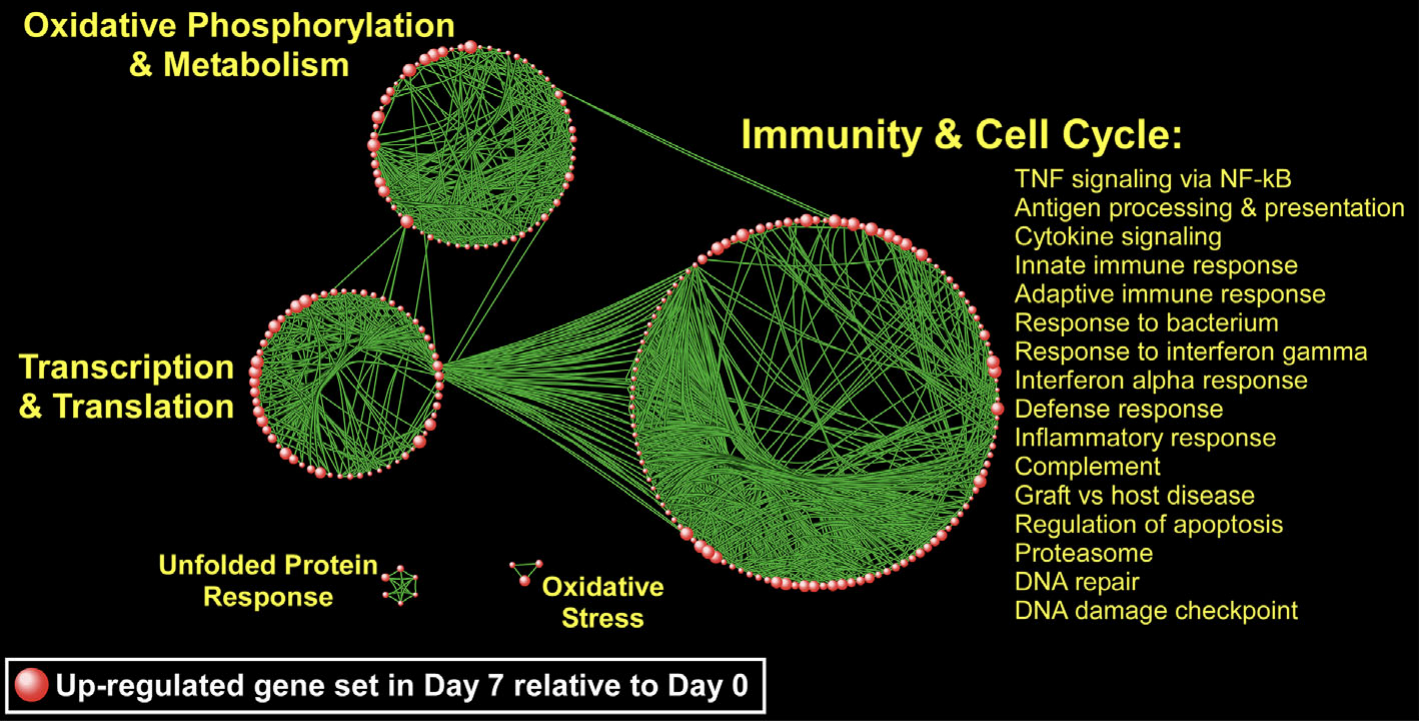
A network-based illustration of enriched monocyte gene sets 1 week after ivacaftor treatment. Each sphere represents an up-regulated gene set and in order to simplify the figure, only the most significantly enriched gene sets are depicted (N = 177, FDR = 0). Connectivity between the pathways is based on 50% or greater overlap among their member genes. The topology of the network is characterized by the emergence of biological modules comprised of highly interconnected gene sets that possess similar functional themes; notable modules include “immunity and cell cycle,” “oxidative phosphorylation and metabolism,” “transcription and translation,” “unfolded protein response,” and “oxidative stress.” representative processes mapping to the most prominent module, “Immunity and Cell Cycle” are shown, and a complete list of all enriched gene sets (FDR < 0.05) is included in **Supplementary Table S2**.

Since genes do not exert their biological influence in isolation (Hartwell et al., 1999), we applied pathway enrichment analyses based on the entire transcriptome using GSEA to further understand the ivacaftor-induced changes in monocyte functional state. We found that treatment with ivacaftor induced an overwhelming up-regulation of cellular pathways in monocytes, with over 1,000 being enriched at FDR < 0.05 (**Table S2**). In contrast, only three gene sets were down-regulated at day 7 (FDR < 0.05) (**Table S3**). This finding indicates that restoring CF function elicits activation of a diverse set of transcriptional programs in peripheral blood monocytes. To visualize the enrichment pattern following ivacaftor therapy, we applied a network-based method to the most significantly up-regulated gene sets (N = 177, FDR = 0). The topology of the resultant network revealed several clusters of highly connected pathways defined as “modules” with distinct biological themes including “immunity and cell cycle,” “oxidative phosphorylation,” “transcription/translation,” “unfolded protein response,” and “Oxidative stress” (**Figure 6**). The largest module was comprised of many immune-related pathways including TNF signaling *via* NF-κB, IFNγ response, inflammatory response, IFNα response, cytokine signaling, and response to bacterium. Collectively, these results indicate that 7 days of ivacaftor treatment in CF subjects alters the functional state of their circulating monocytes by promoting the widespread activation of immuno-inflammatory programs.

## Discussion

Despite many recent advances in CF care, therapies are still needed to dampen baseline chronic CF airway inflammation and to treat enhanced inflammation during CF pulmonary exacerbations. An improved understanding of the molecular and cellular basis of CF inflammation is critical to developing focused anti-inflammatory strategies that will not compromise host defenses or cause significant long-term side effects. Blood monocytes are intriguing therapeutic targets because they can traffic to the inflamed CF airway (Wright et al., 2009; Garratt et al., 2012; Hisert et al., 2019), and they mount aberrant responses in people with CF (del Fresno et al., 2008; Sorio et al., 2015; Hisert et al., 2016), which may contribute to CF disease pathology. Here we use transcriptomics to characterize the phenotypes of peripheral blood monocytes in people with CF before and 7 days after initiation of highly effective modulator therapy with ivacaftor. Our data demonstrate that ivacaftor therapy leads to an rapid change in the transcriptional programming of blood monocytes, predominantly activating genes and transcriptional modules in several broad functional categories, although may are associated with innate immunity and inflammation (**Figure 6**). Ivacaftor also rapidly increased plasma levels of CXCL2 and CCL2, chemokines that summon neutrophils and monocytes respectively. We did not, however, detect changes in several plasma biomarkers that had previously been associated with changes in CF airway inflammation following antibiotic treatment of pulmonary exacerbations.

### Are CFTR Modulators Pro- or Anti-Inflammatory?

From a clinical perspective, ivacaftor appears to decrease inflammation in people with CF: ivacaftor acutely improves lung function and lessens symptoms (Ramsey et al., 2011; Rowe et al., 2014; Hisert et al., 2017), and over time reduces frequency of pulmonary exacerbations (Ramsey et al., 2011; Rowe et al., 2014), diminishes evidence of lung pathology on CT scans (Chassagnon et al., 2016; Hisert et al., 2017; Ronan et al., 2018), and may decrease inflammatory biomarkers in sputum and blood over time (Hisert et al., 2017; Ronan et al., 2018). In addition, macrophages lacking CFTR activity mount hyperinflammatory responses compared to cells with functional CFTR (Bruscia et al., 2009; Bruscia and Bonfield, 2016), and some of these overly exuberant responses are reversed by CFTR modulators (Barnaby et al., 2018; Zhang et al., 2018). It therefore may seem counter-intuitive for restoration of CFTR activity by ivacaftor to enhance multiple monocyte transcriptional pathways associated with inflammation, monocyte expression of canonical inflammatory cytokines, such as TNF and IL-αβ, and plasma levels of chemokines for neutrophils and monocytes. However, studies have shown that *ex vivo* peripheral blood immune cells isolated from people with CF actually exist in an immune-suppressed (or tolerant) state compared to cells from healthy donors. Blood monocytes from people with CF have decreased responses to LPS (del Fresno et al., 2008), and this is thought to be due to exposure of CF monocytes to low levels of LPS that translocate form the CF airway into the plasma (del Campo et al., 2011). Similarly, cells in CF whole blood exposed to multiple toll-like receptor (TLR) agonists mounted less robust inflammatory responses than cells from healthy donors (Kosamo et al., 2019), including decreased secretion of TNF and other NF-κB-induced cytokines.

Is this relative immune-suppressed state of CF peripheral immune cells adaptive and protective, or does it contribute to CF disease pathogenesis? CF subjects with the most robust TLR responses had the most preserved lung function (FEV_1_) and the slowest decline in lung function over time (Kosamo et al., 2019); in other words, impaired peripheral immune cell inflammatory responses in CF are associated with worse lung disease. One explanation for these findings is that impaired immune cell responses in people with CF contribute to airway pathology, and stronger immune responses protect the lung, possibly by fighting bacterial pathogens. Another possibility is that more advanced lung disease results in a leakier barrier between the lungs and the bloodstream, thus exposing peripheral immune cells to higher doses of tolerizing antigens compared to people with less severe lung disease. In support for this second possibility, healthy donor PMBCs cultured in CF plasma experienced marked decreases in transcription compared to cells cultured in healthy donor plasma, and plasma from subjects with severe CF lung disease caused a greater suppression of transcription than plasma from subjects with mild disease (Ideozu et al., 2019). These observations suggest that the *in vivo* plasma milieu in CF can override the hyper-inflammatory influences of intrinsic CFTR dysfunction on innate immune cells’ phenotypes (Murphy and Ribeiro, 2019), and that CFTR modulators therefore potentially exert both pro- and anti-inflammatory effects on immune cells in people with CF.

### Biomarkers That Measure Changes in Inflammation Following Restoration of CFTR Activity May Differ From Biomarkers That Reflect Inflammation Related to Airway Infection

In our prior study of patients with *CFTR-G551D* mutations initiating ivacaftor, we detected no difference in plasma C-reactive protein (CRP) after 1 week of therapy (Hisert et al., 2016). We measured CRP because it is used broadly as a clinical marker of systemic inflammation, and has also been evaluated in a number of studies as a biomarker to detect onset of CF pulmonary exacerbation, efficacy of treatment of CF exacerbations, or severity of CF lung disease (with one parameter for disease severity being whether or not subjects have chronic *P. aeruginosa* infection) (Sharma et al., 2017; Loh et al., 2018; Sagel et al., 2019). In the current study, we evaluated other plasma mediators that have been proposed as biomarkers of inflammation in people with CF. However, many of the analytes tested were below the limit of detection in our assay. We were able to detect high levels of S100A9, IL-1Ra, CXCL2, CCL2, and sCD163 in subject’s plasma pre-ivacaftor. Changes in plasma S100A9, IL-1Ra, and sCD163 have been observed in people with CF who are being treated with antibiotics for pulmonary exacerbations; however, as with CRP, we detected no change in these analytes after 7 days of ivacaftor. These results could reflect that our small cohort was under-powered to detect changes. Alternately, it may be that inflammation caused by an exacerbation/bacteria may be a different phenomenon than inflammation resulting from insufficient CFTR activity. The CF community may need different biomarkers to assess efficacy of CFTR modulators than what are used for measuring onset of exacerbation or efficacy of antibiotics to treat exacerbations.

CXCL2 and CCL2 were both elevated in people with CF compared to healthy controls, and thus the increase in plasma levels of both of these myeloid chemokines after ivacaftor treatment was unexpected. These increases, though, are consistent with our transcriptomic data indicating activation of immuno-inflammatory programs and chemokines in circulating monocytes. Future longitudinal studies of plasma mediators will be important to understand whether there are immediate and delayed changes in plasma inflammatory mediators following restoration of CFTR, and the roles CXCL2 and CCL2 may play in modulating CF airway inflammation.

### Comparisons With Prior CF Immune Cell Transcriptomics Studies

A number of previous studies have characterized the transcriptomes of CF immune cells, and a few have evaluated changes in gene expression following CFTR modulator therapy. To our knowledge, our study is the first report to focus specifically on the transcriptomes of CF monocytes. The most comparable previous study was performed by Sun et al., who characterized the transcriptome of PBMCs collected from subjects enrolled in the GOAL study, a multi-center, prospective characterization of ivacaftor-induced changes in people with *CFTR-G551D* mutations in the USA (Sun et al., 2019). They identified 239 differentially expressed genes (DEGs) when comparing PBMCs pre-ivacaftor and 1 month post-ivacaftor, using a false discovery rate < 0.1; the majority of these genes were decreased in expression after 1 month of ivacaftor treatment. There were no DEGs in common between the GOAL cohort data set, and our data from monocytes isolated pre- and post-ivacaftor. It should be noted that there were many differences between our study and that by Sun et al. In addition to differences in the cell types (PBMCs vs. monocytes) and time points analyzed (1 month vs. 1 week post-ivacaftor), Sun et al. applied a different statistical analysis to their data. We performed a paired analysis, in which all subjects that provided a pre-ivacaftor specimen also provided a post-ivacaftor specimen. Sun et al. included 56 subjects, of which 37 provided both pre- and post-ivacaftor samples, and 19 provided only one sample.

Additionally, the cohort used in the Sun et al. paper differed from our cohort in several important ways. Their cohort (a subset of the total GOAL cohort) (1) had a lower median age than our cohort, (2) was made up of people with *CFTR-G551D* mutations (whereas our cohort all had *CFTR-R117H* mutations), and (3) did not experience a statistically significant increase in FEV_1_ by one month after ivacaftor treatment, when their post-ivacaftor samples were collected. In our cohort, we detected a statistically significant improvement in FEV_1_ by 2 days post ivacaftor that was maintained at day 7, when our post-ivacaftor specimens were collected. The difference in clinical response to ivacaftor may be the key distinction between the two studies. Although the total cohort in the Sun et al. study did not demonstrate a statistically significant improvement in FEV_1_ following ivacaftor treatment, a subset of subjects did experience a clinical response to ivacaftor. Sun et al. distinguished subjects as either “responders” or “non-responders” to ivacaftor based on FEV_1_, body mass index (BMI), and Cystic Fibrosis Questionnaire-Revised (CFQR) respiratory scores (Sun et al., 2019). When we performed functional enrichment analysis on the DEGs with increased expression in the subset of subjects who were “responders” relative to “non-responders” in the Sun et al. cohort, we identified multiple pathways associated with immune function that were significantly up-regulated (**Table S4**). This result suggests that, consistent with our CF cohort findings, peripheral blood immune/inflammatory program activation following initiation of ivacaftor therapy is associated with significant clinical improvements in the GOAL study.

Kopp et al. also characterized immune cell transcriptomes in subjects starting CFTR modulator therapy, and performed both a paired comparison of subjects pre- and post-modulator, and also compared both datasets to transcriptomes from cells from non-CF control subject. Their study examined whole blood from a cohort of delta F508 homozygous subjects, starting lumacaftor/ivacaftor, and they examined a later time point (comparison of pre- and 6 months post-lumacaftor/ivacaftor) (Kopp et al., 2019). In addition to this difference in study design, the clinical response of subjects in this study to lumacaftor/ivacaftor was not as robust, based on change in FEV_1_, as that seen in subjects from studies in which the subjects had ivacaftor-sensitive mutations. Overall, these authors found that blood cells from people with CF both before and after modulator therapy had higher expression of inflammation and apoptosis related genes than cells from healthy donors, and lumacaftor/ivacaftor modestly decreased expression of some inflammatory genes.

In both the studies by Kopp et al. and Sun et al., CFTR modulator treatment was associated with a general dampening of peripheral immune cell inflammatory phenotypes, whereas we found up-regulation of immune and inflammatory transcripts and pathways in blood monocytes one week after initiation of ivacaftor. This difference in trend could be due to the differences in cell populations studied or demographics of the study populations. The choice of time point for assessing changes post initiation of modulator therapy might also be the critical distinction between these studies, indicating that there may be phases of responses to CFTR modulators. CFTR modulators may initially enhance immune cells transcriptional pathways, and then later, when there are decreased airway bacteria and mucous plugging (Hisert et al., 2017), and different or less stimuli in the blood, peripheral blood cell phenotypes may reflect a less inflamed state. As with plasma biomarkers, there are likely both acute and direct effects, as well as the secondary and later effects, of CFTR modulators on immune cells and thus inflammation.

Interpretation of transcriptomics data, particularly when determining whether changes are pro- or anti-inflammatory, is also complicated by the fact that some genes with increased expression may be inhibitors of inflammatory pathways. In fact, several of the most significantly up-regulated genes following ivacaftor treatment in our study are known inhibitors of inflammation. OTUD1 (Ovarian Tumor Family Deubiquitinase 1, FDR = 3.74 × 10^−13^) can inhibit nuclear translocation and transcriptional activity of the IFNγ-activated transcription factor IRF3 (Lu et al., 2018), and loss of function of OTUD1 is associated with auto-immune diseases mediated by interferons (Lu et al., 2018). DUSP2 (Dual Specificity Protein Phosphatase 2, FDR = 1.07 × 10^−10^) belongs to a family of phosphatases that can de-phosphorylate STAT proteins (involved in interferon signaling, in addition to other intracellular signaling cascades), and ERK proteins (involved in toll-like receptor intracellular signaling (Lang et al., 2006; Lu et al., 2015). ATF3 (Activating Transcription Factor 3, FDR = 3.11 × 10^−10^) binds CRE elements in DNA and represses transcription, and has been shown to negatively regulate pro-inflammatory cytokine expression in macrophages (Rosenberger et al., 2008; Labzin et al., 2015). We recently showed that monocytes from the same cohort described here exhibit a decrease in *ex vivo* responsiveness to IFNγ after subjects have received ivacaftor for 7 days (Hisert et al., 2020). An increased expression of genes that deactivate or dampen inflammatory signaling could partly explain why transcriptome data predicted that these monocytes have a more inflammatory phenotype while the same cells, when stimulated *ex vivo*, exhibited decreased IFNγ responses.

### Study Limitations and Strengths

Our study has several limitations. First, our cohort was small, which may have limited our ability to detect important changes in monocyte activation states or plasma biomarkers. Second, we performed our analysis at a single time point after treatment, and thus our data capture an early snapshot of acute changes in monocytes after initiation of ivacaftor, which may not reflect chronic therapy. Most studies evaluating changes in cells and biomarkers following initiation of CFTR modulators have sampled subjects at only one time point, with few longitudinal studies (Rowe et al., 2014; Hisert et al., 2017; Ronan et al., 2018; Kopp et al., 2019; Sun et al., 2019; Harris et al., 2020); however, the synthesis of individual time points with the longitudinal studies suggest that there may be phases of changes in inflammation following restoration of CFTR activity with modulators. The changes reported here occurred within the first week of ivacaftor treatment, and thus reflect acute responses to ivacaftor, and may not reflect a longer term steady-state that incorporates both primary and secondary changes induced by ivacaftor. In future studies of subjects initiating highly active modulator therapy, analyses of changes in inflammatory endpoints at multiple time points should be performed. Third, we did not assess transcriptional profiles of monocytes from healthy control subjects in this study, and therefore we cannot draw conclusions about the relative inflammatory state of CF monocytes compared to those from healthy donors. However, building on the prior literature, we hypothesize that the overall increase in activation of transcriptional programs in CF monocytes post ivacaftor reflects a change from the tolerant state of monocytes pre-ivacaftor, and a return towards the activation state of healthy “wildtype” monocytes following restoration of CFTR activity. Finally, although we investigated an early timepoint to try to detect direct consequences of CFTR restoration by ivacaftor, our data cannot ultimately discriminate whether the changes in monocyte transcriptomes post-ivacaftor are due to direct effects of ivacaftor on monocyte CFTR activity, indirect effects of ivacaftor increasing CFTR activity on other cells in the body (such as airway epithelial cells), or off target effects of ivacaftor on monocytes. The literature suggests that some CF PBMC impairments are likely an indirect consequence of CFTR activity, resulting from exposure of the cells to an altered milieu of CF plasma (Zhang et al., 2019).

Nevertheless, our cohort’s unique design provided several strengths. Our ability to compare cells from the same individuals pre- and post-ivacaftor minimized confounding and maximized statistical power, allowing for detection of statistically significant changes despite the small cohort size. The fact that all subjects were from one institution allowed for processing of specimens on site, and the application of multiple studies to the same specimens, resulting in a deeply phenotyped cohort. Third, we detected a clinically meaningful response to ivacaftor within 48 hours, which provides additional confidence that the changes in immune cells and mediators were related to restoration of CFTR activity and could contribute to changes in airway inflammation. Finally, our data here and in our previous publications describe changes in one immune cell population (monocytes) before and after CFTR modulator therapy, thus providing a detailed functional description of a target cell for potential therapeutic manipulation.

## Summary

Peripheral blood monocytes travel to sites of inflammation, including the CF airway, and are attractive therapeutic targets for dampening the deleterious inflammation in CF lungs. Leveraging an unbiased transcriptomics approach, we systematically characterized changes in the inflammatory phenotypes of these cells following restoration of CFTR activity with ivacaftor. Unexpectedly, our findings revealed that multiple transcriptional programs, including pathways associated with immunity and inflammation, are up-regulated in circulating CF monocytes after one week of ivacaftor treatment. Coincident with this early enhancement of monocyte immuno-inflammatory signals, we identified significant increases in plasma levels of the myeloid chemokines CCL2 and CXCL2 and an overall improvement in FEV_1_. Collectively, our results demonstrate that ivacaftor causes acute alterations in the inflammatory state of blood monocytes in people with CF, which in turn, may modulate airway inflammation and influence lung function. Future studies are necessary to determine if this enhancement of monocyte transcriptional pathways associated with inflammation and immunity is a transient phenomenon, or reflects the new steady state of people with CF receiving highly effective CFTR modulator therapy.

## Data Availability Statement

The datasets presented in this study can be found in online repositories. The names of the repository/repositories and accession number(s) can be found below: https://www.ncbi.nlm.nih.gov/geo/, GSE148076.

## Ethics Statement

The studies involving human participants were reviewed and approved by Research Ethics Committee at St. Vincent’s Hospital, Dublin, Ireland. The patients/participants provided their written informed consent to participate in this study.

## Author Contributions

KH, LB, and EM conceived of the hypothesis and designed the study. KH, TB, KS, BG, SC, and EM acquired and processed specimens. KH, TB, ML, WL, EM, LB, AM, and SG analyzed the data. KH, WL, LB, AM, and SG prepared the manuscript.

## Funding

This study was funded by a CF-Research Innovation Award (200425//210740_6) and an investigator-initiated research grant (ISS-2016-105055) from Vertex Pharmaceuticals, Inc. Funding was also provided by a K08 award (1 K08 HL136786-01A1) and two R01 awards (R01 AI137111 and R01 HL152724) from the National Institutes of Health.

## Conflict of Interest

The authors declare that the research was conducted in the absence of any commercial or financial relationships that could be construed as a potential conflict of interest.
