# Supplementary material for: CFTR Modulator Therapy Enhances Peripheral Blood Monocyte Contributions to Immune Responses in People With Cystic Fibrosis
Source: Front Pharmacol. 2020 Aug 13;11:1219. doi: 10.3389/fphar.2020.01219 (PMC7461946; doi:10.3389/fphar.2020.01219)
Supplement: Supplementary file 1 [file DataSheet_1.pdf]

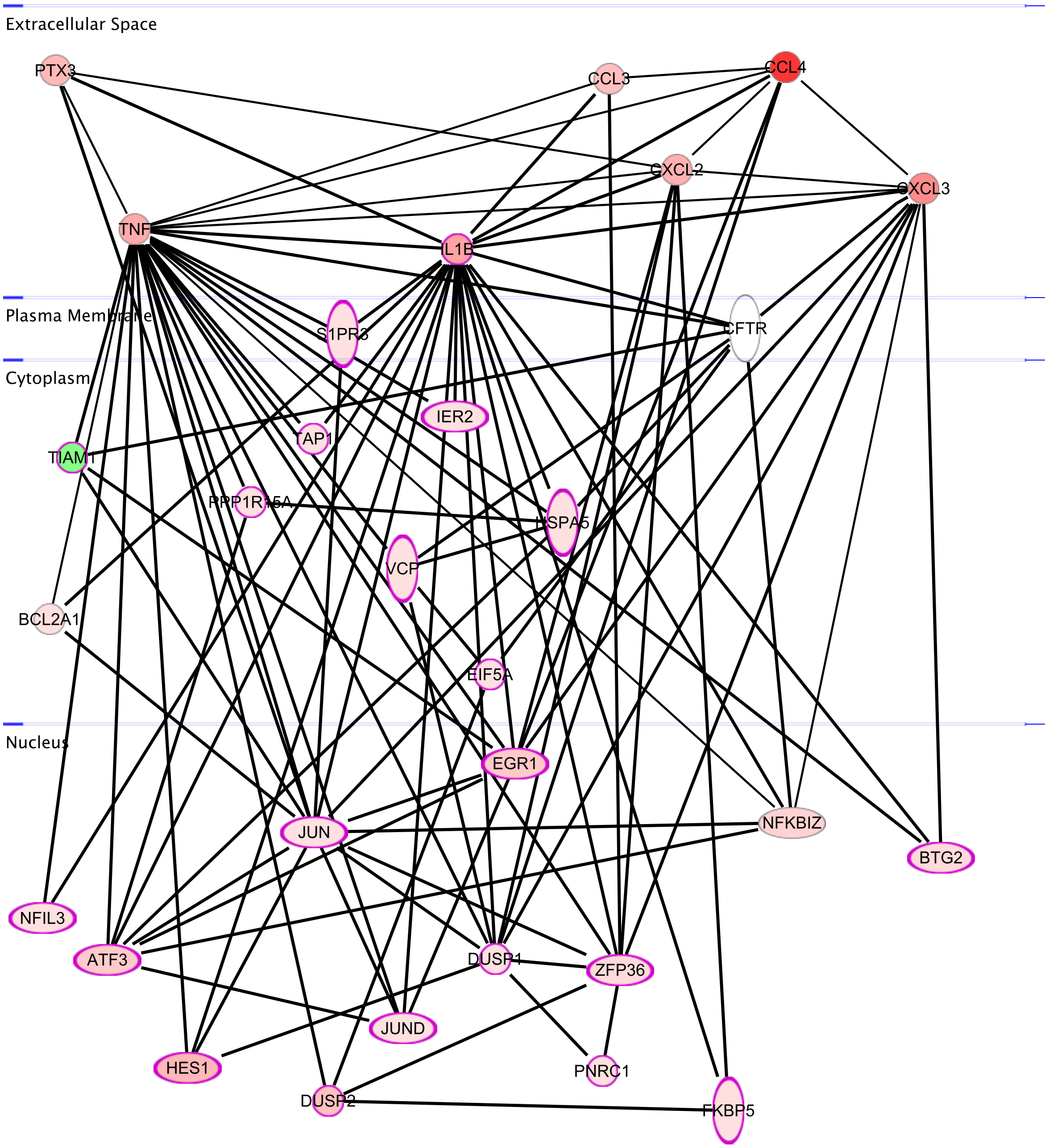

Figure S1: Gene product interaction network analysis of differentially expressed monocyte genes after 1 week of ivacaftor therapy with all nodes labeled. This "interactome" was constructed based on known relationships among the differentially expressed genes. To depict interaction of these nodes with the target of ivacaftor, we also added CFTR as a seed to the network. Hubs that are up-regulated depicted as red spheres, and those down regulated depicted. as green spheres.

Table S1. List of differentially expressed genes (FDR < 0.05) in monocytes of CF subjects before treatment with ivacaftor (D0) and day 7 post ivacaftor (D7)

Ensembl_ID	Gene Symbol	Description	Log_2[D7/D0]	P-value	FDR
ENSG00000165312	OTUD1	OTU deubiquitinase 1	0.6563	2.77E-17	3.74E-13
ENSG00000158050	DUSP2	dual specificity phosphatase 2	1.0573	1.59E-14	1.07E-10
ENSG00000162772	ATF3	activating transcription factor 3	0.8722	6.91E-14	3.11E-10
ENSG00000164691	TAGAP	T-cell activation RhoGTPase activating protein	0.4554	2.05E-11	6.93E-08
ENSG00000159388	BTG2	BTG family, member 2	0.6095	3.51E-10	9.47E-07
ENSG00000125538	IL1B	interleukin 1, beta	1.5618	1.49E-09	3.36E-06
ENSG00000232810	TNF	tumor necrosis factor	1.4257	1.98E-08	3.81E-05
ENSG00000146278	PNRC1	proline-rich nuclear receptor coactivator 1	0.5111	7.45E-08	1.26E-04
ENSG00000154099	DNAAF1	dynein, axonemal, assembly factor 1	2.6599	1.08E-07	1.33E-04
ENSG00000162783	IER5	immediate early response 5	0.5101	1.00E-07	1.33E-04
ENSG00000163734	CXCL3	chemokine (C-X-C motif) ligand 3	2.1813	8.87E-08	1.33E-04
ENSG00000147416	ATP6V1B2	ATPase, H+ transporting, lysosomal 56/58kDa, V1 subunit B2	0.2907	1.33E-07	1.50E-04
ENSG00000044574	HSPA5	heat shock 70kDa protein 5 (glucose-regulated protein, 78kDa)	0.3651	1.94E-07	2.02E-04
ENSG00000120738	EGR1	early growth response 1	0.9069	2.65E-07	2.55E-04
ENSG00000163661	PTX3	pentraxin 3, long	1.1676	3.54E-07	3.19E-04
ENSG00000114315	HES1	hes family bHLH transcription factor 1	1.1437	6.07E-07	5.13E-04
ENSG00000177606	JUN	jun proto-oncogene	0.4911	7.44E-07	5.91E-04
ENSG00000160888	IER2	immediate early response 2	0.5048	1.45E-06	1.09E-03
ENSG00000275302	CCL4	chemokine (C-C motif) ligand 4	4.0443	4.01E-06	2.85E-03
ENSG00000128016	ZFP36	ZFP36 ring finger protein	0.6332	6.52E-06	4.40E-03
ENSG00000129534	MIS18BP1	MIS18 binding protein 1	-0.2422	7.30E-06	4.70E-03
ENSG00000277632	CCL3	chemokine (C-C motif) ligand 3	1.1136	7.82E-06	4.80E-03
ENSG00000168394	TAP1	transporter 1, ATP-binding cassette, sub-family B (MDR/TAP)	0.3573	9.00E-06	5.28E-03
ENSG00000096060	FKBP5	FK506 binding protein 5	0.4496	1.30E-05	7.32E-03
ENSG00000081041	CXCL2	chemokine (C-X-C motif) ligand 2	1.3774	1.42E-05	7.67E-03
ENSG00000120129	DUSP1	dual specificity phosphatase 1	0.3256	1.95E-05	1.01E-02
ENSG00000067208	EVI5	ecotropic viral integration site 5	-0.3220	2.85E-05	1.33E-02
ENSG00000087074	PPP1R15A	protein phosphatase 1, regulatory subunit 15A	0.4241	2.76E-05	1.33E-02
ENSG00000276070	CCL4L1	chemokine (C-C motif) ligand 4-like 1	3.3671	2.80E-05	1.33E-02
ENSG00000169442	CD52	CD52 molecule	0.2944	3.48E-05	1.57E-02
ENSG00000271614	LINC00936	long intergenic non-protein coding RNA 936	0.4255	4.07E-05	1.77E-02
ENSG00000156299	TIAM1	T-cell lymphoma invasion and metastasis 1	-0.2215	4.46E-05	1.88E-02
ENSG00000130522	JUND	jun D proto-oncogene	0.4404	4.81E-05	1.97E-02
ENSG00000165030	NFIL3	nuclear factor, interleukin 3 regulated	0.5019	5.03E-05	2.00E-02
ENSG00000090924	PLEKHG2	pleckstrin homology domain containing, family G (with RhoGef domain) member 2	0.6430	5.53E-05	2.13E-02
ENSG00000178996	SNX18	sorting nexin 18	-0.2034	6.73E-05	2.53E-02
ENSG00000258738	AL121603.2	novel transcript, antisense to BAZ1A	0.8496	8.97E-05	3.27E-02
ENSG00000144802	NFKBIZ	nuclear factor of kappa light polypeptide gene enhancer in B-cells inhibitor, zeta	0.7228	9.71E-05	3.45E-02
ENSG00000213694	S1PR3	sphingosine-1-phosphate receptor 3	0.4020	1.23E-04	4.26E-02
ENSG00000110917	MLEC	malectin	0.2884	1.34E-04	4.34E-02
ENSG00000132507	EIF5A	eukaryotic translation initiation factor 5A	0.2424	1.31E-04	4.34E-02
ENSG00000180596	HIST1H2BC	histone cluster 1, H2bc	0.9006	1.35E-04	4.34E-02
ENSG00000131174	COX7B	cytochrome c oxidase subunit VIIb	0.3143	1.43E-04	4.48E-02
ENSG00000140379	BCL2A1	BCL2-related protein A1	0.4717	1.49E-04	4.58E-02
ENSG00000165280	VCP	valosin containing protein	0.2356	1.58E-04	4.73E-02
ENSG00000163867	ZMYM6	zinc finger, MYM-type 6	-0.3773	1.62E-04	4.76E-02
ENSG00000169508	GPR183	G protein-coupled receptor 183	0.9544	1.69E-04	4.78E-02
ENSG00000273033	LINC02035	long intergenic non-protein coding RNA 2035	-0.3506	1.70E-04	4.78E-02
ENSG00000163041	H3F3A	H3 histone, family 3A	-0.3813	1.81E-04	4.99E-02

Table S2. GSEA list of differentially up-regulated processes (FDR < 0.05) in monocytes of CF subjects after treatment with ivacaftor for 7 days.

Gene Set Name	Number of Genes	FDR
HALLMARK_OXIDATIVE_PHOSPHORYLATION	198	0
KEGG_RIBOSOME	86	0
GO_PROTEIN_LOCALIZATION_TO_ENDOPLASMIC_RETICULUM	118	0
REACTOME_METABOLISM_OF_PROTEINS	394	0
KEGG_OXIDATIVE_PHOSPHORYLATION	102	0
GO_ESTABLISHMENT_OF_PROTEIN_LOCALIZATION_TO_ENDOPLASMIC_RETICULUM	102	0
GO_CYTOSOLIC_RIBOSOME	105	0
REACTOME_SRP_DEPENDENT_COTRANSLATIONAL_PROTEIN_TARGETING_TO_MEMBRANE	119	0
REACTOME_INFLUENZA_VIRAL_RNA_TRANSCRIPTION_AND_REPLICATION	111	0
REACTOME_PEPTIDE_CHAIN_ELONGATION	95	0
GO_TRANSLATIONAL_INITIATION	141	0
REACTOME_METABOLISM_OF_MRNA	219	0
HALLMARK_TNFA_SIGNALING_VIA_NFKB	193	0
REACTOME_NONSENSE_MEDIATED_DECAY_ENHANCED_BY_THE_EXON_JUNCTION_COMPLEX	116	0
GO_RIBOSOMAL_SUBUNIT	156	0
REACTOME_TRANSLATION	157	0
GO_RIBOSOME	212	0
KEGG_PARKINSONS_DISEASE	99	0
GO_NUCLEAR_TRANSCRIBED_MRNA_CATABOLIC_PROCESS_NONSENSE_MEDIATED_DECAY	117	0
REACTOME_3_UTR_MEDIATED_TRANSLATIONAL_REGULATION	116	0
GO_CYTOSOLIC_PART	197	0
REACTOME_TCA_CYCLE_AND_RESPIRATORY_ELECTRON_TRANSPORT	113	0
GO_MITOCHONDRIAL_PROTEIN_COMPLEX	126	0
REACTOME_RESPIRATORY_ELECTRON_TRANSPORT_ATP_SYNTHESIS_BY_CHEMIOSMOTIC_COUPLING_AND_HEAT_PRODUCTION_BY_UNCOUPLING_PROTEINS_	78	0
GO_STRUCTURAL_CONSTITUENT_OF_RIBOSOME	194	0
GO_NUCLEOSIDE_TRIPHOSPHATE_METABOLIC_PROCESS	193	0
GO_INNER_MITOCHONDRIAL_MEMBRANE_PROTEIN_COMPLEX	98	0
GO_CELLULAR_RESPONSE_TO_CYTOKINE_STIMULUS	475	0
REACTOME_INFLUENZA_LIFE_CYCLE	145	0
REACTOME_METABOLISM_OF_RNA	264	0
GO_MITOCHONDRIAL_MEMBRANE_PART	154	0
GO_AMIDE_BIOSYNTHETIC_PROCESS	460	0
GO_ESTABLISHMENT_OF_PROTEIN_LOCALIZATION_TO_ORGANELLE	333	0
GO_ORGANELLE_INNER_MEMBRANE	454	0
GO_NUCLEOSIDE_MONOPHOSPHATE_METABOLIC_PROCESS	206	0
GO_MULTI_ORGANISM_METABOLIC_PROCESS	136	0
GO_PROTEIN_TARGETING_TO_MEMBRANE	146	0
GO_PROTEIN_TARGETING	367	0
GO_ESTABLISHMENT_OF_PROTEIN_LOCALIZATION_TO_MEMBRANE	243	0
GO_CYTOKINE_MEDIATED_SIGNALING_PATHWAY	351	0
KEGG_ALZHEIMERS_DISEASE	139	0
GO_PROTEIN_LOCALIZATION_TO_ORGANELLE	497	0
GO_GLYCOSYL_COMPOUND_METABOLIC_PROCESS	310	0
GO_CELL_SUBSTRATE_JUNCTION	352	0
GO_NUCLEOBASE_CONTAINING_SMALL_MOLECULE_METABOLIC_PROCESS	441	0
REACTOME_RESPIRATORY_ELECTRON_TRANSPORT	63	0
GO_RNA_CATABOLIC_PROCESS	214	0
HALLMARK_MYC_TARGETS_V1	198	0
GO_OXIDATIVE_PHOSPHORYLATION	77	0
GO_CYTOSOLIC_LARGE_RIBOSOMAL_SUBUNIT	57	0
HALLMARK_INTERFERON_GAMMA_RESPONSE	197	0
GO_LARGE_RIBOSOMAL_SUBUNIT	90	0
KEGG_HUNTINGTONS_DISEASE	154	0
GO_VIRAL_LIFE_CYCLE	264	0
GO_PROTON_TRANSPORTING_TWO_SECTOR_ATPASE_COMPLEX	42	0
GO_ORGANIC_CYCLIC_COMPOUND_CATABOLIC_PROCESS	363	0
GO_PURINE_CONTAINING_COMPOUND_METABOLIC_PROCESS	320	0
GO_GENERATION_OF_PRECURSOR_METABOLITES_AND_ENERGY	241	0
GO_HYDROGEN_ION_TRANSMEMBRANE_TRANSPORT	82	0
GO_RRNA_METABOLIC_PROCESS	249	0
GO_RIBOSOME_BIOGENESIS	300	0
GO_CELLULAR_RESPIRATION	129	0
GO_ANTIGEN_PROCESSING_AND_PRESENTATION_OF_PEPTIDE_ANTIGEN	162	0
GO_INNATE_IMMUNE_RESPONSE	468	0
GO_ELECTRON_TRANSPORT_CHAIN	88	0

GO_MITOCHONDRIAL_MATRIX	369	0
GO_INFLAMMATORY_RESPONSE	353	0
GO_ANTIGEN_PROCESSING_AND_PRESENTATION_OF_PEPTIDE_ANTIGEN_VIA_MHC_CLASS_I	88	0
GO_ANCHORING_JUNCTION	418	0
REACTOME_ER_PHAGOSOME_PATHWAY	59	0
GO_ANTIGEN_PROCESSING_AND_PRESENTATION	192	0
GO_RESPIRATORY_CHAIN	72	0
GO_PROTEIN_LOCALIZATION_TO_MEMBRANE	330	0
GO_ENERGY_DERIVATION_BY_OXIDATION_OF_ORGANIC_COMPOUNDS	184	0
GO_RIBONUCLEOPROTEIN_COMPLEX_BIOGENESIS	420	0
GO_MYELIN_SHEATH	145	0
REACTOME_ADAPTIVE_IMMUNE_SYSTEM	480	0
GO_HYDROGEN_TRANSPORT	109	0
GO_SMALL_RIBOSOMAL_SUBUNIT	66	0
REACTOME_SIGNALING_BY_WNT	62	0
GO_MITOCHONDRIAL_TRANSMEMBRANE_TRANSPORT	52	0
GO_LUMENAL_SIDE_OF_MEMBRANE	30	0
GO_POSITIVE_REGULATION_OF_IMMUNE_RESPONSE	491	0
GO_PIGMENT_GRANULE	93	0
GO_RESPONSE_TO_BACTERIUM	385	0
REACTOME_ANTIGEN_PROCESSING_CROSS_PRESENTATION	73	0
GO_MITOCHONDRIAL_TRANSPORT	160	0
GO_CYTOSOLIC_SMALL_RIBOSOMAL_SUBUNIT	42	0
GO_ANTIGEN_PROCESSING_AND_PRESENTATION_OF_EXOGENOUS_PEPTIDE_ANTIGEN_VIA_MHC_CLASS_I	65	0
KEGG_ANTIGEN_PROCESSING_AND_PRESENTATION	64	0
GO_STRUCTURAL_MOLECULE_ACTIVITY	474	0
HALLMARK_MTORC1_SIGNALING	197	0
GO_NADH_DEHYDROGENASE_COMPLEX	42	0
GO_ACTIVATION_OF_IMMUNE_RESPONSE	376	0
GO_PROTON_TRANSPORTING_ATP_SYNTHASE_COMPLEX	22	0
REACTOME_FORMATION_OF_THE_TERNARY_COMPLEX_AND_SUBSEQUENTLY_THE_43S_COMPLEX	55	0
GO_CELLULAR_COMPONENT_DISASSEMBLY	430	0
GO_RESPONSE_TO_INTERFERON_GAMMA	121	0
GO_POSTTRANSCRIPTIONAL_REGULATION_OF_GENE_EXPRESSION	394	0
GO_POSITIVE_REGULATION_OF_DEFENSE_RESPONSE	310	0
REACTOME_SCFSKP2_MEDIATED_DEGRADATION_OF_P27_P21	53	0
GO_IMMUNE_EFFECTOR_PROCESS	398	0
GO_TRANSLATIONAL_ELONGATION	108	0
GO_NEGATIVE_REGULATION_OF_TRANSFERASE_ACTIVITY	286	0
HALLMARK_INFLAMMATORY_RESPONSE	172	0
GO_ENDOSOMAL_PART	377	0
GO_DEFENSE_RESPONSE_TO_OTHER_ORGANISM	349	0
GO_MITOCHONDRIAL_ATP_SYNTHESIS_COUPLED_PROTON_TRANSPORT	17	0
GO_OXIDOREDUCTASE_COMPLEX	86	0
REACTOME_ACTIVATION_OF_THE_MRNA_UPON_BINDING_OF_THE_CAP_BINDING_COMPLEX_AND_EIFS_AND_SUBSEQUENT_BINDING_TO_43S	63	0
GO_CELLULAR_RESPONSE_TO_INTERFERON_GAMMA	102	0
GO_ACTIVATION_OF_INNATE_IMMUNE_RESPONSE	191	0
GO_IMMUNE_RESPONSE_REGULATING_CELL_SURFACE_RECEPTOR_SIGNALING_PATHWAY	293	0
REACTOME_VIF_MEDIATED_DEGRADATION_OF_APOBEC3G	49	0
KEGG_PROTEASOME	43	0
REACTOME_CYCLIN_E_ASSOCIATED_EVENTS_DURING_G1_S_TRANSITION_	62	0
HALLMARK_UV_RESPONSE_UP	142	0
HALLMARK_ALLOGRAFT_REJECTION	185	0
REACTOME_AUTODEGRADATION_OF_THE_E3_UBIQUITIN_LIGASE_COP1	47	0
GO_POSITIVE_REGULATION_OF_INNATE_IMMUNE_RESPONSE	229	0
GO_PROTEASOME_COMPLEX	75	0
GO_ANTIGEN_BINDING	86	0
HALLMARK_ADIPOGENESIS	181	0
GO_PROTEIN_FOLDING	194	0
GO_REGULATION_OF_INNATE_IMMUNE_RESPONSE	316	0
GO_HYDROGEN_EXPORTING_ATPASE_ACTIVITY	24	0
GO_RRNA_BINDING	56	0
REACTOME_DESTABILIZATION_OF_MRNA_BY_AUF1_HNRNP_D0	50	0
REACTOME_FORMATION_OF_ATP_BY_CHEMIOSMOTIC_COUPLING	13	0
GO_ENDOCYTIC_VESICLE_MEMBRANE	120	0
GO_INTERFERON_GAMMA_MEDIATED_SIGNALING_PATHWAY	66	0
GO_MITOCHONDRIAL_RESPIRATORY_CHAIN_COMPLEX_ASSEMBLY	63	0
REACTOME_P53_DEPENDENT_G1_DNA_DAMAGE_RESPONSE	53	0

HALLMARK_INTERFERON_ALPHA_RESPONSE	94	0
GO_FC_RECEPTOR_SIGNALING_PATHWAY	191	0
GO_REGULATION_OF_PROTEIN_MODIFICATION_BY_SMALL_PROTEIN_CONJUGATION_OR_REMOVAL	260	0
GO_RESPONSE_TO_TOPOLOGICALLY_INCORRECT_PROTEIN	148	0
REACTOME_SCF_BETA_TRCP_MEDIATED_DEGRADATION_OF_EMI1	49	0
REACTOME_CDK_MEDIATED_PHOSPHORYLATION_AND_REMOVAL_OF_CDC6	46	0
GO_REGULATION_OF_CYTOKINE_PRODUCTION	476	0
GO_VESICLE_MEMBRANE	400	0
GO_CYTOPLASMIC_VESICLE_PART	454	0
GO_TRANSPORT_VESICLE	256	0
HALLMARK_UNFOLDED_PROTEIN_RESPONSE	109	0
REACTOME_CLASS_I_MHC_MEDIATED_ANTIGEN_PROCESSING_PRESENTATION	222	0
GO_INNATE_IMMUNE_RESPONSE_ACTIVATING_CELL_SURFACE_RECEPTOR_SIGNALING_PATHWAY	102	0
GO_NCRNA_PROCESSING	369	0
REACTOME_REGULATION_OF_ORNITHINE_DECARBOXYLASE_ODC	48	0
GO_UNFOLDED_PROTEIN_BINDING	87	0
GO_NIK_NF_KAPPAB_SIGNALING	79	0
GO_MACROMOLECULAR_COMPLEX_DISASSEMBLY	170	0
GO_OXIDOREDUCTASE_ACTIVITY_ACTING_ON_NAD_P_H_QUINONE_OR_SIMILAR_COMPOUND_AS_ACCEPTOR	49	0
REACTOME_ACTIVATION_OF_NF_KAPPAB_IN_B_CELLS	61	0
GO_TUMOR_NECROSIS_FACTOR_MEDIATED_SIGNALING_PATHWAY	107	0
REACTOME_CYTOKINE_SIGNALING_IN_IMMUNE_SYSTEM	242	0
HALLMARK_COMPLEMENT	176	0
GO_COATED_VESICLE	202	0
REACTOME_P53_INDEPENDENT_G1_S_DNA_DAMAGE_CHECKPOINT	48	0
REACTOME_CROSS_PRESENTATION_OF_SOLUBLE_EXOGENOUS_ANTIGENS_ENDOSOMES	47	0
GO_INTRINSIC_COMPONENT_OF_ORGANELLE_MEMBRANE	230	0
GO_AUTOPHAGY	323	0
GO_SIDE_OF_MEMBRANE	345	0
GO_LYTIC_VACUOLE	442	0
GO_TRANSMEMBRANE_RECEPTOR_PROTEIN_TYROSINE_KINASE_SIGNALING_PATHWAY	383	0
GO_POSITIVE_REGULATION_OF_PROTEOLYSIS	328	0
REACTOME_METABOLISM_OF_CARBOHYDRATES	177	0
REACTOME_REGULATION_OF_APOPTOSIS	54	0
GO_REGULATION_OF_MULTI_ORGANISM_PROCESS	383	0
KEGG_GRAFT_VERSUS_HOST_DISEASE	36	0
GO_REGULATION_OF_CELLULAR_AMINO_ACID_METABOLIC_PROCESS	57	0
GO_POSITIVE_REGULATION_OF_LIGASE_ACTIVITY	103	0
REACTOME_HIV_INFECTION	191	0
GO_COATED_VESICLE_MEMBRANE	119	0
GO_ANAPHASE_PROMOTING_COMPLEX_DEPENDENT_CATABOLIC_PROCESS	75	0
GO_MITOCHONDRIAL_RESPIRATORY_CHAIN_COMPLEX_I_BIOGENESIS	51	0
REACTOME_AUTODEGRADATION_OF_CDH1_BY_CDH1_APC_C	56	0
GO_CELLULAR_PROTEIN_COMPLEX_DISASSEMBLY	118	0
REACTOME_CDT1_ASSOCIATION_WITH_THE_CDC6_ORC_ORIGIN_COMPLEX	54	6.35E-06
GO_CELLULAR_PROTEIN_COMPLEX_ASSEMBLY	290	6.38E-06
GO_OXIDOREDUCTASE_ACTIVITY_ACTING_ON_NAD_P_H	84	6.41E-06
GO_DEFENSE_RESPONSE_TO_BACTERIUM	143	6.44E-06
GO_CATION_TRANSPORTING_ATPASE_ACTIVITY	49	6.47E-06
REACTOME_PROTEIN_FOLDING	46	6.50E-06
GO_POSITIVE_REGULATION_OF_PROTEIN_CATABOLIC_PROCESS	241	6.53E-06
KEGG_TYPE_I_DIABETES_MELLITUS	36	6.56E-06
GO_PROTEIN_TARGETING_TO_MITOCHONDRION	47	6.60E-06
KEGG_PENTOSE_PHOSPHATE_PATHWAY	23	6.63E-06
KEGG_VIBRIO_CHOLERAE_INFECTION	44	6.66E-06
GO_RIBOSOME_ASSEMBLY	49	6.69E-06
KEGG_PYRIMIDINE_METABOLISM	89	6.72E-06
GO_RESPONSE_TO_TUMOR_NECROSIS_FACTOR	187	6.76E-06
REACTOME_APOPTOSIS	134	6.79E-06
REACTOME_HOST_INTERACTIONS_OF_HIV_FACTORS	122	6.82E-06
REACTOME_SIGNALING_BY_THE_B_CELL_RECEPTOR_BCR	121	6.86E-06
GO_NEGATIVE_REGULATION_OF_INTRACELLULAR_SIGNAL_TRANSDUCTION	352	6.89E-06
GO_PEPTIDE_ANTIGEN_BINDING	26	6.93E-06
GO_RESPONSE_TO_MOLECULE_OF_BACTERIAL_ORIGIN	271	6.96E-06
GO_REGULATION_OF_IMMUNE_EFFECTOR_PROCESS	358	7.00E-06
GO_REGULATION_OF_PROTEIN_CATABOLIC_PROCESS	352	7.03E-06
REACTOME_MEMBRANE_TRAFFICKING	104	7.07E-06
REACTOME_APC_C_CDC20_MEDIATED_DEGRADATION_OF_MITOTIC_PROTEINS	65	7.11E-06

REACTOME_INTERFERON_GAMMA_SIGNALING	59	7.14E-06
GO_REGULATION_OF_PROTEIN_UBIQUITINATION_INVOLVED_IN_UBIQUITIN_DEPENDENT_PROTEIN_CATABOLIC_PROCESS	98	7.18E-06
GO_REGULATION_OF_PEPTIDASE_ACTIVITY	278	7.22E-06
REACTOME_PREFOLDIN_MEDIATED_TRANSFER_OF_SUBSTRATE_TO_CCT_TRIC	22	7.26E-06
GO_RESPONSE_TO_ENDOPLASMIC_RETICULUM_STRESS	213	7.29E-06
GO_LYTIC_VACUOLE_MEMBRANE	242	7.33E-06
GO_REGULATION_OF_SYMBIOSIS_ENCOMPASSING_MUTUALISM_THROUGH_PARASITISM	179	7.37E-06
GO_RESPONSE_TO_PEPTIDE	293	7.41E-06
REACTOME_DOWNSTREAM_SIGNALING_EVENTS_OF_B_CELL_RECEPTOR_BCR	92	7.45E-06
GO_TRANSLATIONAL_TERMINATION	92	7.49E-06
GO_ATP_HYDROLYSIS_COUPLED_TRANSMEMBRANE_TRANSPORT	29	7.53E-06
GO_NEGATIVE_REGULATION_OF_PROTEIN_MODIFICATION_BY_SMALL_PROTEIN_CONJUGATION_OR_REMOVAL	130	7.57E-06
GO_REGULATION_OF_APOPTOTIC_SIGNALING_PATHWAY	307	7.62E-06
GO_PROTON_TRANSPORTING_TWO_SECTOR_ATPASE_COMPLEX_CATALYTIC_DOMAIN	15	7.66E-06
GO_RESPONSE_TO_TYPE_I_INTERFERON	54	7.70E-06
GO_MHC_PROTEIN_COMPLEX	24	7.74E-06
REACTOME_GLYCOLYSIS	23	1.23E-05
HALLMARK_APOPTOSIS	150	1.23E-05
KEGG_LEISHMANIA_INFECTION	68	1.24E-05
REACTOME_REGULATION_OF_MRNA_STABILITY_BY_PROTEINS_THAT_BIND_AU_RICH_ELEMENTS	81	1.25E-05
GO_REGULATION_OF_CELLULAR_PROTEIN_LOCALIZATION	468	1.25E-05
GO_REGULATION_OF_RNA_STABILITY	129	1.79E-05
REACTOME_INTERFERON_SIGNALING	140	1.80E-05
GO_IRE1_MEDIATED_UNFOLDED_PROTEIN_RESPONSE	55	1.80E-05
GO_REGULATION_OF_LIGASE_ACTIVITY	122	1.81E-05
GO_CARBOHYDRATE_BINDING	199	1.82E-05
KEGG_ALLOGRAFT_REJECTION	31	1.83E-05
GO_PROTEASOMAL_PROTEIN_CATABOLIC_PROCESS	258	2.34E-05
GO_CARBOHYDRATE_METABOLIC_PROCESS	487	2.35E-05
GO_POSITIVE_REGULATION_OF_ESTABLISHMENT_OF_PROTEIN_LOCALIZATION	406	2.36E-05
GO_PROTEIN_MATURATION	216	2.37E-05
REACTOME_ANTIGEN_PRESENTATION_FOLDING_ASSEMBLY_AND_PEPTIDE_LOADING_OF_CLASS_I_MHC	21	2.38E-05
GO_REGULATION_OF_MAPK_CASCADE	485	2.92E-05
GO_GLYCOSYL_COMPOUND_BIOSYNTHETIC_PROCESS	105	3.31E-05
GO_NON_CANONICAL_WNT_SIGNALING_PATHWAY	116	3.32E-05
REACTOME_APC_C_CDH1_MEDIATED_DEGRADATION_OF_CDC20_AND_OTHER_APC_C_CDH1_TARGETED_PROTEINS_IN_LATE_MITOSIS_EARLY_G1	64	3.34E-05
REACTOME_FORMATION_OF_TUBULIN_FOLDING_INTERMEDIATES_BY_CCT_TRIC	16	3.35E-05
GO_RESPONSE_TO_OXIDATIVE_STRESS	299	3.36E-05
REACTOME_G1_S_TRANSITION	104	3.38E-05
GO_MITOCHONDRIAL_ELECTRON_TRANSPORT_CYTOCHROME_C_TO_OXYGEN	14	3.39E-05
GO_RESPONSE_TO_INTERLEUKIN_1	87	3.41E-05
GO_CELLULAR_HOMEOSTASIS	490	3.42E-05
HALLMARK_P53_PATHWAY	186	3.43E-05
GO_ANTIGEN_RECEPTOR_MEDIATED_SIGNALING_PATHWAY	178	3.45E-05
GO_NUCLEOSIDE_TRIPHOSPHATE_BIOSYNTHETIC_PROCESS	53	3.46E-05
GO_ENDOCYTOSIS	418	3.48E-05
BIOCARTA_GLYCOLYSIS_PATHWAY	8	3.49E-05
GO_REGULATION_OF_ESTABLISHMENT_OF_PLANAR_POLARITY	97	3.51E-05
REACTOME_UNFOLDED_PROTEIN_RESPONSE	74	3.80E-05
BIOCARTA_ERK_PATHWAY	24	3.82E-05
GO_ENERGY_COUPLED_PROTON_TRANSPORT_DOWN_ELECTROCHEMICAL_GRADIENT	22	3.83E-05
GO_ER_TO_GOLGI_TRANSPORT_VESICLE	64	3.85E-05
REACTOME_ACTIVATION_OF_CHAPERONE_GENES_BY_XBP1S	44	4.19E-05
GO_REGULATION_OF_CELL_ACTIVATION	407	4.21E-05
GO_GTPASE_ACTIVITY	209	4.22E-05
GO_ER_TO_GOLGI_TRANSPORT_VESICLE_MEMBRANE	50	4.24E-05
REACTOME_INTERFERON_ALPHA_BETA_SIGNALING	50	4.26E-05
GO_RESPONSE_TO_HYDROGEN_PEROXIDE	98	4.27E-05
GO_RIBONUCLEOSIDE_TRIPHOSPHATE_BIOSYNTHETIC_PROCESS	41	4.29E-05
GO_AMIDE_BINDING	202	4.31E-05
GO_POSITIVE_REGULATION_OF_ORGANELLE_ORGANIZATION	486	4.32E-05
GO_EARLY_ENDOSOME	253	4.66E-05
GO_ISOMERASE_ACTIVITY	138	4.68E-05
GO_IRON_ION_TRANSPORT	44	4.70E-05
GO_ATP_BIOSYNTHETIC_PROCESS	30	6.11E-05
GO_PROTON_TRANSPORTING_TWO_SECTOR_ATPASE_COMPLEX_PROTON_TRANSPORTING_DOMAIN	19	6.13E-05
REACTOME_ORC1_REMOVAL_FROM_CHROMATIN	65	6.15E-05
GO_POSITIVE_REGULATION_OF_CELL_ACTIVATION	264	6.18E-05

GO_PROTON_TRANSPORTING_ATP_SYNTHASE_COMPLEX_COUPLING_FACTOR_F_O_	11	6.20E-05
GO_T_CELL_RECEPTOR_SIGNALING_PATHWAY	137	7.03E-05
HALLMARK_IL2_STAT5_SIGNALING	184	7.05E-05
HALLMARK_FATTY_ACID_METABOLISM	137	7.08E-05
GO_RESPONSE_TO_REACTIVE_OXYGEN_SPECIES	161	7.11E-05
GO_POSITIVE_REGULATION_OF_PROTEIN_MODIFICATION_BY_SMALL_PROTEIN_CONJUGATION_OR_REMOVAL	181	7.36E-05
GO_COFACTOR_METABOLIC_PROCESS	283	7.39E-05
REACTOME_REGULATION_OF_MITOTIC_CELL_CYCLE	77	7.42E-05
GO_LEUKOCYTE_MEDIATED_IMMUNITY	150	7.44E-05
GO_RESPONSE_TO_TOXIC_SUBSTANCE	188	7.47E-05
REACTOME_INSULIN_RECEPTOR_RECYCLING	17	7.50E-05
PID_P38_MK2_PATHWAY	20	8.20E-05
REACTOME_MHC_CLASS_II_ANTIGEN_PRESENTATION	80	8.23E-05
REACTOME_ASSEMBLY_OF_THE_PRE_REPLICATIVE_COMPLEX	63	8.26E-05
GO_REGULATION_OF_PROTEIN_BINDING	147	8.29E-05
REACTOME_POST_TRANSLATIONAL_PROTEIN_MODIFICATION	145	8.32E-05
GO_POSITIVE_REGULATION_OF_CATABOLIC_PROCESS	352	8.65E-05
REACTOME_SYNTHESIS_OF_DNA	89	9.58E-05
GO_PURINE_NUCLEOSIDE_BIOSYNTHETIC_PROCESS	78	1.05E-04
GO_POSITIVE_REGULATION_OF_CANONICAL_WNT_SIGNALING_PATHWAY	97	1.09E-04
REACTOME_METABOLISM_OF_NUCLEOTIDES	63	1.10E-04
GO_POSITIVE_REGULATION_OF_CYTOKINE_PRODUCTION	313	1.11E-04
GO_REGULATION_OF_ORGAN_MORPHOGENESIS	169	1.12E-04
GO_LATE_ENDOSOME_MEMBRANE	90	1.12E-04
GO_CARBOHYDRATE_BIOSYNTHETIC_PROCESS	96	1.12E-04
GO_NUCLEOSIDE_PHOSPHATE_BIOSYNTHETIC_PROCESS	153	1.13E-04
GO_PHAGOSOME_MATURATION	30	1.13E-04
GO_ORGANELLAR_RIBOSOME	68	1.14E-04
GO_REGULATION_OF_CELLULAR_AMINE_METABOLIC_PROCESS	72	1.15E-04
GO_PHAGOSOME_ACIDIFICATION	21	1.18E-04
GO_CELLULAR_RESPONSE_TO_INSULIN_STIMULUS	114	1.18E-04
GO_TRIVALENT_INORGANIC_CATION_TRANSPORT	30	1.19E-04
GO_ORGANONITROGEN_COMPOUND_CATABOLIC_PROCESS	258	1.19E-04
GO_REGULATION_OF_RESPONSE_TO_WOUNDING	304	1.20E-04
GO_CELL_ACTIVATION	473	1.45E-04
REACTOME_S_PHASE	105	1.49E-04
KEGG_LYSOSOME	114	1.57E-04
GO_CELLULAR_RESPONSE_TO_TOPOLOGICALLY_INCORRECT_PROTEIN	111	1.57E-04
GO_MIDBRAIN_DEVELOPMENT	62	1.65E-04
BIOCARTA_MPR_PATHWAY	27	1.69E-04
GO_CELLULAR_RESPONSE_TO_NITROGEN_COMPOUND	366	1.73E-04
GO_TRANSITION_METAL_ION_TRANSPORT	82	1.73E-04
REACTOME_M_G1_TRANSITION	76	1.80E-04
GO_CYTOCHROME_COMPLEX	15	1.81E-04
GO_CARBOHYDRATE_DERIVATIVE_BIOSYNTHETIC_PROCESS	455	1.84E-04
GO_LATE_ENDOSOME	186	2.00E-04
REACTOME_GLUCOSE_METABOLISM	56	2.01E-04
GO_RESPONSE_TO_INSULIN	157	2.13E-04
GO_ENDOPLASMIC_RETICULUM_LUMEN	141	2.16E-04
GO_PURINE_NUCLEOSIDE_MONOPHOSPHATE_BIOSYNTHETIC_PROCESS	55	2.17E-04
GO_POSITIVE_REGULATION_OF_LOCOMOTION	319	2.19E-04
GO_OXIDOREDUCTION_COENZYME_METABOLIC_PROCESS	87	2.20E-04
GO_NEGATIVE_REGULATION_OF_CANONICAL_WNT_SIGNALING_PATHWAY	127	2.27E-04
GO_GLYCERALDEHYDE_3_PHOSPHATE_METABOLIC_PROCESS	16	2.30E-04
GO_PHAGOCYTOSIS	163	2.30E-04
HALLMARK_DNA_REPAIR	141	2.54E-04
GO_NEGATIVE_REGULATION_OF_IMMUNE_RESPONSE	97	2.55E-04
GO_REGULATION_OF_CELLULAR_AMIDE_METABOLIC_PROCESS	303	2.56E-04
GO_OXIDOREDUCTASE_ACTIVITY_ACTING_ON_PEROXIDE_AS_ACCEPTOR	32	2.57E-04
GO_PROTON_TRANSPORTING_V_TYPE_ATPASE_COMPLEX	18	2.58E-04
GO_MHC_PROTEIN_COMPLEX_BINDING	17	2.67E-04
KEGG_AUTOIMMUNE_THYROID_DISEASE	30	2.71E-04
GO_NUCLEOSIDE_MONOPHOSPHATE_BIOSYNTHETIC_PROCESS	73	2.78E-04
GO_PROTEIN_LOCALIZATION_TO_MITOCHONDRION	65	2.85E-04
GO_PROTEIN_OLIGOMERIZATION	331	2.93E-04
GO_GLYCOSPHINGOLIPID_METABOLIC_PROCESS	51	3.04E-04
SA_TRKA_RECEPTOR	15	3.07E-04
GO_FC_EPSILON_RECEPTOR_SIGNALING_PATHWAY	130	3.39E-04

GO_REGULATION_OF_CELLULAR_PROTEIN_CATABOLIC_PROCESS	251	3.42E-04
GO_INTRINSIC_COMPONENT_OF_ENDOPLASMIC_RETICULUM_MEMBRANE	114	3.57E-04
GO_ELECTRON_CARRIER_ACTIVITY	98	3.67E-04
GO_METHYLOSOME	12	3.68E-04
GO_MHC_CLASS_I_PROTEIN_COMPLEX	10	3.82E-04
REACTOME_MRNA_SPLICING_MINOR_PATHWAY	41	4.16E-04
GO_ORGANELLE_SUBCOMPARTMENT	271	4.18E-04
GO_RESPONSE_TO_FIBROBLAST_GROWTH_FACTOR	75	4.27E-04
GO_RESPONSE_TO_WOUNDING	414	4.38E-04
PID_MYC_ACTIV_PATHWAY	75	4.61E-04
GO_NEGATIVE_REGULATION_OF_CYTOKINE_PRODUCTION	176	4.63E-04
GO_REGULATION_OF_ACTIN_CYTOSKELETON_REORGANIZATION	25	4.76E-04
KEGG_GLYCOLYSIS_GLUCONEOGENESIS	40	4.78E-04
GO_PROTEASOME_ACCESSORY_COMPLEX	24	4.79E-04
HALLMARK_HYPOXIA	161	4.86E-04
PID_AP1_PATHWAY	55	4.88E-04
REACTOME_MITOTIC_G1_G1_S_PHASES	129	4.92E-04
GO_POSITIVE_REGULATION_OF_CELLULAR_PROTEIN_CATABOLIC_PROCESS	178	4.93E-04
GO_POSITIVE_REGULATION_OF_CELLULAR_PROTEIN_LOCALIZATION	301	4.93E-04
GO_SMALL_MOLECULE_BIOSYNTHETIC_PROCESS	353	4.94E-04
GO_MONOCARBOXYLIC_ACID_METABOLIC_PROCESS	368	4.95E-04
GO_POSITIVE_REGULATION_OF_MAPK_CASCADE	340	5.26E-04
BIOCARTA_PROTEASOME_PATHWAY	28	5.31E-04
GO_NEGATIVE_REGULATION_OF_APOPTOTIC_SIGNALING_PATHWAY	162	5.33E-04
GO_G_PROTEIN_ALPHA_SUBUNIT_BINDING	17	5.87E-04
GO_OXIDOREDUCTASE_ACTIVITY_ACTING_ON_A_HEME_GROUP_OF_DONORS	20	6.16E-04
GO_POSITIVE_REGULATION_OF_APOPTOTIC_SIGNALING_PATHWAY	155	6.24E-04
GO_POSITIVE_REGULATION_OF_PEPTIDASE_ACTIVITY	135	6.25E-04
REACTOME_MITOCHONDRIAL_PROTEIN_IMPORT	49	6.52E-04
GO_REGULATION_OF_BINDING	240	6.65E-04
GO_REGULATION_OF_CYSTEINE_TYPE_ENDOPEPTIDASE_ACTIVITY	186	6.86E-04
HALLMARK_IL6_JAK_STAT3_SIGNALING	78	6.88E-04
KEGG_CARDIAC_MUSCLE_CONTRACTION	49	7.40E-04
GO_PURINE_CONTAINING_COMPOUND_BIOSYNTHETIC_PROCESS	113	7.42E-04
GO_GUANYL_NUCLEOTIDE_BINDING	315	7.90E-04
REACTOME_DIABETES_PATHWAYS	109	8.28E-04
REACTOME_ASPARAGINE_N_LINKED_GLYCOSYLATION	75	8.37E-04
BIOCARTA_SALMONELLA_PATHWAY	13	8.39E-04
GO_CYTOPLASMIC_TRANSLATION	41	8.99E-04
GO_ANTIGEN_PROCESSING_AND_PRESENTATION_OF_ENDOGENOUS_ANTIGEN	16	9.00E-04
GO_PENTOSE_PHOSPHATE_SHUNT	12	9.05E-04
GO_MONOSACCHARIDE_METABOLIC_PROCESS	144	9.11E-04
KEGG_PURINE_METABOLISM	131	9.13E-04
GO_REGULATION_OF_PROTEIN_SECRETION	289	9.52E-04
GO_ANTIGEN_PROCESSING_AND_PRESENTATION_OF_PEPTIDE_OR_POLYSACCHARIDE_ANTIGEN_VIA_MHC_CLASS_II	83	9.64E-04
KEGG_PRION_DISEASES	31	9.65E-04
KEGG_EPITHELIAL_CELL_SIGNALING_IN_HELICOBACTER_PYLORI_INFECTION	56	9.80E-04
GO_POSITIVE_REGULATION_OF_INTRACELLULAR_TRANSPORT	301	1.00E-03
GO_MITOCHONDRIAL_MEMBRANE_ORGANIZATION	89	1.05E-03
GO_SPLICEOSOMAL_TRI_SNRNP_COMPLEX	26	1.07E-03
GO_GOLGI_ASSOCIATED_VESICLE	70	1.07E-03
GO_REGULATION_OF_CYTOKINE_SECRETION	116	1.07E-03
GO_REGULATION_OF_INFLAMMATORY_RESPONSE	221	1.07E-03
GO_POSITIVE_REGULATION_OF_LEUKOCYTE_CHEMOTAXIS	66	1.07E-03
GO_PROTEASE_BINDING	86	1.10E-03
KEGG_SPLICEOSOME	124	1.11E-03
GO_CELLULAR_RESPONSE_TO_PEPTIDE	203	1.12E-03
GO_CELL_CHEMOTAXIS	129	1.13E-03
PID_HIF2PATHWAY	30	1.14E-03
GO_REGULATION_OF_MAP_KINASE_ACTIVITY	259	1.14E-03
GO_REGULATION_OF_TELOMERASE_RNA_LOCALIZATION_TO_CAJAL_BODY	15	1.16E-03
GO_ENDOCYTIC_VESICLE	213	1.21E-03
GO_COPI_COATED_VESICLE	22	1.21E-03
GO_MHC_CLASS_II_PROTEIN_COMPLEX	14	1.21E-03
GO_REGULATION_OF_CANONICAL_WNT_SIGNALING_PATHWAY	184	1.21E-03
GO_LEUKOCYTE_CHEMOTAXIS	94	1.22E-03
GO_RIBOSOMAL_SMALL_SUBUNIT_BIOGENESIS	57	1.23E-03
GO_RESPONSE_TO_INORGANIC_SUBSTANCE	361	1.23E-03

GO_NEGATIVE_REGULATION_OF_WNT_SIGNALING_PATHWAY	150	1.27E-03
GO_SMALL_MOLECULE_CATABOLIC_PROCESS	260	1.27E-03
GO_REGULATION_OF_CELLULAR_KETONE_METABOLIC_PROCESS	132	1.27E-03
REACTOME_LATENT_INFECTION_OF_HOMO_SAPIENS_WITH_MYCOBACTERIUM_TUBERCULOSIS	25	1.32E-03
GO_SULFUR_COMPOUND_METABOLIC_PROCESS	274	1.32E-03
GO_THREONINE_TYPE_PEPTIDASE_ACTIVITY	19	1.32E-03
GO_RIBONUCLEOPROTEIN_COMPLEX_SUBUNIT_ORGANIZATION	181	1.33E-03
GO_REGULATION_OF_PROTEIN_SERINE_THREONINE_KINASE_ACTIVITY	392	1.36E-03
REACTOME_SIGNALLING_BY_NGF	195	1.40E-03
GO_COENZYME_METABOLIC_PROCESS	222	1.41E-03
GO_POSITIVE_REGULATION_OF_CELL_ADHESION	298	1.41E-03
REACTOME_ANTIGEN_PROCESSING_UBIQUITINATION_PROTEASOME_DEGRADATION	184	1.44E-03
KEGG_PATHOGENIC_ESCHERICHIA_COLI_INFECTION	48	1.48E-03
GO_RESPONSE_TO_GROWTH_FACTOR	341	1.54E-03
GO_LEUKOCYTE_MIGRATION	217	1.55E-03
GO_CELLULAR_RESPONSE_TO_INTERLEUKIN_4	24	1.56E-03
GO_PROTON_TRANSPORTING_ATP_SYNTHASE_ACTIVITY_ROTATIONAL_MECHANISM	11	1.57E-03
GO_NEGATIVE_REGULATION_OF_RESPONSE_TO_WOUNDING	109	1.58E-03
KEGG_VIRAL_MYOCARDITIS	55	1.60E-03
GO_ANGIOGENESIS	209	1.60E-03
GO_REGULATION_OF_LEUKOCYTE_DIFFERENTIATION	194	1.61E-03
GO_RESPONSE_TO_VIRUS	202	1.64E-03
GO_ORGANELLE_ENVELOPE_LUMEN	72	1.64E-03
GO_NEGATIVE_REGULATION_OF_TRANSPORT	336	1.67E-03
GO_REGULATION_OF_TRANSLATIONAL_INITIATION	77	1.69E-03
GO_NEGATIVE_REGULATION_OF_IMMUNE_SYSTEM_PROCESS	306	1.70E-03
GO_LEUKOCYTE_ACTIVATION	353	1.77E-03
GO_GLUCOSE_6_PHOSPHATE_METABOLIC_PROCESS	18	1.77E-03
REACTOME_MRNA_SPLICING	105	1.94E-03
GO_RESPONSE_TO_MECHANICAL_STIMULUS	146	1.97E-03
GO_PROTEIN_EXIT_FROM_ENDOPLASMIC_RETICULUM	19	1.97E-03
REACTOME_KERATAN_SULFATE_KERATIN_METABOLISM	18	1.99E-03
GO_NAD_METABOLIC_PROCESS	44	1.99E-03
GO_REGULATION_OF_SMOOTH_MUSCLE_CELL_PROLIFERATION	78	1.99E-03
REACTOME_CELL_CYCLE_CHECKPOINTS	108	1.99E-03
GO_ATPASE_COUPLED_ION_TRANSMEMBRANE_TRANSPORTER_ACTIVITY	58	1.99E-03
REACTOME_TRANSFERRIN_ENDOCYTOSIS_AND_RECYCLING	20	1.99E-03
GO_SIGNAL_TRANSDUCTION_BY_PROTEIN_PHOSPHORYLATION	322	2.01E-03
BIOCARTA_CDK5_PATHWAY	10	2.01E-03
GO_KERATAN_SULFATE_METABOLIC_PROCESS	21	2.06E-03
GO_CHAPERONE_MEDIATED_PROTEIN_FOLDING	45	2.08E-03
GO_ANTIGEN_PROCESSING_AND_PRESENTATION_OF_ENDOGENOUS_PEPTIDE_ANTIGEN	13	2.10E-03
GO_ENDOPLASMIC_RETICULUM_CHAPERONE_COMPLEX	10	2.11E-03
GO_POSITIVE_REGULATION_OF_CHEMOTAXIS	91	2.12E-03
GO_NUCLEOTIDE_PHOSPHORYLATION	47	2.13E-03
REACTOME_HEMOSTASIS	368	2.14E-03
GO_RIBOSOMAL_SMALL_SUBUNIT_ASSEMBLY	17	2.14E-03
GO_DEFENSE_RESPONSE_TO_FUNGUS	22	2.16E-03
GO_PROTEIN_CHANNEL_ACTIVITY	11	2.17E-03
GO_REGULATION_OF_INTERFERON_GAMMA_PRODUCTION	80	2.19E-03
GO_PHAGOCYTIC_VESICLE_MEMBRANE	53	2.21E-03
GO_CELLULAR_RESPONSE_TO_OXIDATIVE_STRESS	163	2.24E-03
GO_CELLULAR_RESPONSE_TO_BIOTIC_STIMULUS	148	2.24E-03
GO_ENDOPLASMIC_RETICULUM_TO_CYTOSOL_TRANSPORT	20	2.27E-03
BIOCARTA_INTEGRIN_PATHWAY	35	2.31E-03
GO_AMINOGLYCAN_METABOLIC_PROCESS	117	2.32E-03
GO_INTRINSIC_COMPONENT_OF_MITOCHONDRIAL_MEMBRANE	44	2.32E-03
GO_CLATHRIN_COATED_VESICLE_MEMBRANE	65	2.32E-03
GO_PYRUVATE_METABOLIC_PROCESS	46	2.33E-03
GO_SERINE_TYPE_CARBOXYPEPTIDASE_ACTIVITY	10	2.33E-03
GO_ER_ASSOCIATED_UBIQUITIN_DEPENDENT_PROTEIN_CATABOLIC_PROCESS	60	2.33E-03
GO_AEROBIC_RESPIRATION	47	2.34E-03
GO_MUCOPOLYSACCHARIDE_METABOLIC_PROCESS	76	2.37E-03
GO_CELLULAR_RESPONSE_TO_LIPID	350	2.37E-03
GO_CELLULAR_RESPONSE_TO_INTERLEUKIN_1	64	2.38E-03
GO_POSITIVE_REGULATION_OF_WNT_SIGNALING_PATHWAY	123	2.40E-03
GO_RAGE_RECEPTOR_BINDING	10	2.40E-03
GO_POSITIVE_REGULATION_OF_MITOCHONDRION_ORGANIZATION	145	2.43E-03

GO_CLATHRIN_COATED_ENDOCYTIC_VESICLE_MEMBRANE	38	2.45E-03
GO_REGULATION_OF_MITOCHONDRION_ORGANIZATION	190	2.45E-03
GO_RIBOSOMAL_LARGE_SUBUNIT_BIOGENESIS	46	2.56E-03
GO_EXTERNAL_SIDE_OF_PLASMA_MEMBRANE	179	2.70E-03
PID_PDGFRB_PATHWAY	126	2.72E-03
GO_RECEPTOR_MEDIATED_ENDOCYTOSIS	171	2.73E-03
KEGG_ASTHMA	21	2.78E-03
GO_SUBSTANTIA_NIGRA_DEVELOPMENT	40	2.78E-03
GO_OUTER_MEMBRANE	162	2.78E-03
NABA_MATRISOME_ASSOCIATED	389	2.80E-03
GO_REGULATION_OF_HEMOPOIESIS	265	2.90E-03
GO_CYTOKINE_BINDING	69	2.90E-03
GO_NEGATIVE_REGULATION_OF_ENDOPLASMIC_RETICULUM_UNFOLDED_PROTEIN_RESPONSE	10	2.90E-03
GO_MONOVALENT_INORGANIC_CATION_TRANSPORT	266	2.91E-03
GO_RIBOSOMAL_LARGE_SUBUNIT_ASSEMBLY	21	3.06E-03
GO_MHC_CLASS_II_PROTEIN_COMPLEX_BINDING	15	3.11E-03
GO_ION_CHANNEL_BINDING	82	3.12E-03
GO_REGULATION_OF_I_KAPPAB_KINASE_NF_KAPPAB_SIGNALING	214	3.15E-03
GO_RETROGRADE_PROTEIN_TRANSPORT_ER_TO_CYTOSOL	15	3.19E-03
GO_NEGATIVE_REGULATION_OF_MULTI_ORGANISM_PROCESS	124	3.28E-03
GO_MONOSACCHARIDE_CATABOLIC_PROCESS	47	3.28E-03
GO_TRANSLATION_ELONGATION_FACTOR_ACTIVITY	19	3.28E-03
GO_U12_TYPE_SPLICEOSOMAL_COMPLEX	25	3.29E-03
GO_NEGATIVE_REGULATION_OF_DEFENSE_RESPONSE	114	3.30E-03
GO_NEGATIVE_REGULATION_OF_PROTEIN_BINDING	69	3.31E-03
HALLMARK_GLYCOLYSIS	171	3.31E-03
GO_DETOXIFICATION	58	3.48E-03
GO_REGULATION_OF_HOMEOSTATIC_PROCESS	339	3.58E-03
GO_SEQUESTERING_OF_METAL_ION	8	3.62E-03
HALLMARK_PEROXISOME	91	3.70E-03
GO_HEXOSE_METABOLIC_PROCESS	126	3.70E-03
REACTOME_GLYCOSAMINOGLYCAN_METABOLISM	72	3.76E-03
GO_VESICLE_COAT	42	3.76E-03
GO_CYTOKINE_ACTIVITY	121	3.77E-03
GO_POSITIVE_REGULATION_OF_LEUKOCYTE_MIGRATION	90	3.77E-03
GO_CELLULAR_CHEMICAL_HOMEOSTASIS	397	3.85E-03
GO_HEXOSE_CATABOLIC_PROCESS	38	3.85E-03
GO_POSITIVE_REGULATION_OF_PROTEIN_TARGETING_TO_MEMBRANE	15	4.04E-03
GO_REGULATION_OF_VIRAL_ENTRY_INTO_HOST_CELL	21	4.08E-03
GO_ERAD_PATHWAY	73	4.08E-03
GO_PATTERN_RECOGNITION_RECEPTOR_SIGNALING_PATHWAY	101	4.11E-03
GO_PROTEIN_TETRAMERIZATION	109	4.33E-03
REACTOME_GLYCOSPHINGOLIPID_METABOLISM	29	4.33E-03
REACTOME_SIGNALING_BY_INSULIN_RECEPTOR	80	4.37E-03
GO_ATP_GENERATION_FROM_ADP	29	4.37E-03
GO_INTRINSIC_COMPONENT_OF_MITOCHONDRIAL_OUTER_MEMBRANE	21	4.50E-03
GO_NEGATIVE_REGULATION_OF_IMMUNE_EFFECTOR_PROCESS	86	4.70E-03
GO_CELLULAR_RESPONSE_TO_HORMONE_STIMULUS	405	4.71E-03
GO_LIPOPOLYSACCHARIDE_MEDIATED_SIGNALING_PATHWAY	31	4.72E-03
GO_REGULATION_OF_MITOCHONDRIAL_MEMBRANE_POTENTIAL	49	4.72E-03
GO_PLASMA_MEMBRANE_PROTEIN_COMPLEX	332	4.79E-03
REACTOME_IMMUNOREGULATORY_INTERACTIONS_BETWEEN_A_LYMPHOID_AND_A_NON_LYMPHOID_CELL	57	4.80E-03
GO_NADH_METABOLIC_PROCESS	29	4.82E-03
GO_ARP2_3_PROTEIN_COMPLEX	11	4.82E-03
GO_REGENERATION	115	4.83E-03
GO_MEMBRANE_MICRODOMAIN	230	4.83E-03
REACTOME_METABOLISM_OF_AMINO_ACIDS_AND_DERIVATIVES	154	4.84E-03
GO_BINDING_OF_SPERM_TO_ZONA_PELLUCIDA	19	4.85E-03
GO_AMINOGLYCAN_BIOSYNTHETIC_PROCESS	72	4.86E-03
GO_WOUND_HEALING	344	4.88E-03
GO_HUMORAL_IMMUNE_RESPONSE	120	5.00E-03
GO_TRANS_GOLGI_NETWORK_TRANSPORT_VESICLE	25	5.01E-03
GO_NEGATIVE_REGULATION_OF_INTRINSIC_APOPTOTIC_SIGNALING_PATHWAY	75	5.03E-03
REACTOME_IRON_UPTAKE_AND_TRANSPORT	30	5.06E-03
GO_REGULATION_OF_RESPONSE_TO_BIOTIC_STIMULUS	192	5.09E-03
GO_REGULATION_OF_DEFENSE_RESPONSE_TO_VIRUS	154	5.10E-03
GO_INSULIN_RECEPTOR_SIGNALING_PATHWAY	63	5.12E-03
GO_EPHRIN_RECEPTOR_SIGNALING_PATHWAY	70	5.15E-03

GO_MONOCYTE_CHEMOTAXIS	26	5.16E-03
GO_ORGAN_REGENERATION	64	5.29E-03
GO_REGULATION_OF_LEUKOCYTE_PROLIFERATION	176	5.45E-03
GO_FC_GAMMA_RECEPTOR_SIGNALING_PATHWAY	83	5.45E-03
GO_LIPID_PARTICLE	56	5.46E-03
GO_RESPONSE_TO_EXTRACELLULAR_STIMULUS	315	5.47E-03
GO_REGULATION_OF_CYTOKINE_PRODUCTION_INVOLVED_IN_IMMUNE_RESPONSE	54	5.50E-03
KEGG_NATURAL_KILLER_CELL_MEDIATED_CYTOTOXICITY	105	5.50E-03
GO_RESPONSE_TO_INTERLEUKIN_4	28	5.55E-03
GO_ACTIVATION_OF_CYSTEINE_TYPE_ENDOPEPTIDASE_ACTIVITY	89	5.71E-03
GO_REGULATION_OF_INTERLEUKIN_6_PRODUCTION	87	5.71E-03
PID_UPA_UPAR_PATHWAY	30	5.97E-03
GO_CARBOHYDRATE_DERIVATIVE_CATABOLIC_PROCESS	138	6.03E-03
GO_LEUKOCYTE_MEDIATED_CYTOTOXICITY	27	6.04E-03
GO_REGULATION_OF_LEUKOCYTE_MIGRATION	119	6.09E-03
REACTOME_EXTRACELLULAR_MATRIX_ORGANIZATION	47	6.09E-03
GO_CELL_PROLIFERATION	492	6.09E-03
GO_CELLULAR_RESPONSE_TO_EXTERNAL_STIMULUS	207	6.13E-03
GO_SERINE_TYPE_EXOPEPTIDASE_ACTIVITY	12	6.14E-03
GO_NF_KAPPAB_BINDING	30	6.19E-03
GO_GRANULOCYTE_MIGRATION	55	6.22E-03
GO_NEGATIVE_REGULATION_OF_ESTABLISHMENT_OF_PROTEIN_LOCALIZATION	165	6.23E-03
PID_CASPASE_PATHWAY	51	6.23E-03
GO_REGULATION_OF_VASCULATURE_DEVELOPMENT	165	6.24E-03
GO_ADAPTIVE_IMMUNE_RESPONSE	229	6.24E-03
GO_CARBOHYDRATE_CATABOLIC_PROCESS	87	6.25E-03
GO_REGULATION_OF_CELL_ADHESION	497	6.38E-03
BIOCARTA_IGF1_PATHWAY	20	6.41E-03
GO_NEURAL_NUCLEUS_DEVELOPMENT	46	6.60E-03
GO_CALCIUM_DEPENDENT_PROTEIN_BINDING	46	6.66E-03
GO_REGULATION_OF_INTRINSIC_APOPTOTIC_SIGNALING_PATHWAY	124	6.68E-03
GO_ORGANELLAR_LARGE_RIBOSOMAL_SUBUNIT	31	6.73E-03
GO_REGULATION_OF_CALCIUM_ION_TRANSPORT_INTO_CYTOSOL	60	6.82E-03
GO_LIPOSACCHARIDE_METABOLIC_PROCESS	91	6.83E-03
GO_NEGATIVE_REGULATION_OF_MAP_KINASE_ACTIVITY	60	6.99E-03
GO_REGULATION_OF_ACUTE_INFLAMMATORY_RESPONSE	57	7.04E-03
GO_MONOVALENT_INORGANIC_CATION_TRANSMEMBRANE_TRANSPORTER_ACTIVITY	205	7.04E-03
PID_ERBB1_DOWNSTREAM_PATHWAY	103	7.05E-03
GO_CELLULAR_LIPID_CATABOLIC_PROCESS	116	7.13E-03
GO_POSITIVE_REGULATION_OF_BINDING	106	7.15E-03
GO_POSITIVE_REGULATION_OF_ACTIN_CYTOSKELETON_REORGANIZATION	12	7.25E-03
GO_REGULATION_OF_LEUKOCYTE_CHEMOTAXIS	77	7.26E-03
GO_NEGATIVE_REGULATION_OF_VIRAL_PROCESS	78	7.27E-03
GO_ZYMOGEN_ACTIVATION	95	7.27E-03
GO_REGULATION_OF_RESPONSE_TO_EXTRACELLULAR_STIMULUS	154	7.27E-03
GO_MITOCHONDRIAL_TRANSLATION	105	7.28E-03
GO_CELLULAR_RESPONSE_TO_ARSENIC_CONTAINING_SUBSTANCE	14	7.31E-03
GO_HEAT_SHOCK_PROTEIN_BINDING	82	7.33E-03
GO_CYTOKINE_RECEPTOR_BINDING	179	7.39E-03
GO_INORGANIC_ION_TRANSMEMBRANE_TRANSPORT	348	7.54E-03
PID_CD8_TCR_DOWNSTREAM_PATHWAY	52	7.55E-03
GO_RESPONSE_TO_UV_B	14	7.55E-03
GO_PH_REDUCTION	32	7.57E-03
GO_MULTI_ORGANISM_MEMBRANE_ORGANIZATION	29	7.61E-03
GO_TAXIS	322	7.78E-03
GO_MITOCHONDRIAL_ELECTRON_TRANSPORT_UBIQUINOL_TO_CYTOCHROME_C	12	7.83E-03
GO_REGULATION_OF_RELEASE_OF_SEQUESTERED_CALCIUM_ION_INTO_CYTOSOL	50	7.85E-03
GO_MONOSACCHARIDE_BINDING	59	7.87E-03
GO_COPI_VESICLE_COAT	12	8.09E-03
REACTOME_NGF_SIGNALLING_VIA_TRKA_FROM_THE_PLASMA_MEMBRANE	121	8.30E-03
GO_NEURON_DEATH	37	8.42E-03
GO_GLUCOSE_METABOLIC_PROCESS	95	8.47E-03
GO_NEGATIVE_REGULATION_OF_PHOSPHORYLATION	332	8.48E-03
GO_REGULATION_OF_ERBB_SIGNALING_PATHWAY	68	8.55E-03
GO_SULFUR_COMPOUND_BIOSYNTHETIC_PROCESS	155	8.69E-03
BIOCARTA_SRCRPTP_PATHWAY	10	8.81E-03
GO_REACTIVE_OXYGEN_SPECIES_METABOLIC_PROCESS	72	8.83E-03
GO_SPHINGOLIPID_METABOLIC_PROCESS	110	8.90E-03

GO_POSITIVE_REGULATION_OF_ACTIN_FILAMENT_POLYMERIZATION	59	8.91E-03
GO_REGULATION_OF_HOMOTYPIC_CELL_CELL_ADHESION	264	8.92E-03
GO_REGULATION_OF_WNT_SIGNALING_PATHWAY	236	9.09E-03
GO_REGULATION_OF_MORPHOGENESIS_OF_A_BRANCHING_STRUCTURE	26	9.10E-03
GO_APOPTOTIC_SIGNALING_PATHWAY	262	9.26E-03
GO__DE_NOVO_PROTEIN_FOLDING	18	9.30E-03
GO_POSITIVE_REGULATION_OF_PRODUCTION_OF_MOLECULAR_MEDIATOR_OF_IMMUNE_RESPONSE	55	9.37E-03
GO_COPI_COATED_VESICLE_MEMBRANE	16	9.48E-03
GO_POSITIVE_REGULATION_OF_RESPONSE_TO_EXTERNAL_STIMULUS	224	9.54E-03
GO_REGULATION_OF_ERK1_AND_ERK2_CASCADE	158	9.54E-03
GO_VIRION_ASSEMBLY	35	9.74E-03
GO_INACTIVATION_OF_MAPK_ACTIVITY	22	9.79E-03
GO_MESODERM_DEVELOPMENT	78	9.99E-03
BIOCARTA_MTA3_PATHWAY	15	1.00E-02
GO_LEUKOCYTE_CELL_CELL_ADHESION	215	1.01E-02
GO_NEGATIVE_REGULATION_OF_KINASE_ACTIVITY	195	1.01E-02
GO_ION_HOMEOSTASIS	400	1.01E-02
GO_SMOOTH_ENDOPLASMIC_RETICULUM	25	1.01E-02
GO_PHOSPHOLIPASE_INHIBITOR_ACTIVITY	8	1.01E-02
GO_TOXIN_TRANSPORT	34	1.01E-02
GO_GLYCOLIPID_CATABOLIC_PROCESS	10	1.01E-02
REACTOME_SIGNALLING_TO_RAS	24	1.01E-02
GO_POSITIVE_REGULATION_OF_PROTEIN_SECRETION	156	1.01E-02
GO_CELLULAR_RESPONSE_TO_ATP	11	1.01E-02
GO_REGULATION_OF_CALCIUM_ION_IMPORT	66	1.02E-02
KEGG_N_GLYCAN_BIOSYNTHESIS	44	1.02E-02
REACTOME_SIGNAL_TRANSDUCTION_BY_L1	32	1.02E-02
GO_POSITIVE_REGULATION_OF_PROTEIN_COMPLEX_ASSEMBLY	168	1.02E-02
GO_CLATHRIN_COATED_VESICLE	130	1.02E-02
GO_POSITIVE_REGULATION_OF_CYTOSKELETON_ORGANIZATION	145	1.02E-02
REACTOME_POST_CHAPERONIN_TUBULIN_FOLDING_PATHWAY	13	1.04E-02
GO_NEGATIVE_REGULATION_OF_NF_KAPPAB_IMPORT_INTO_NUCLEUS	16	1.05E-02
PID_IL2_1PATHWAY	54	1.06E-02
KEGG_CELL_ADHESION_MOLECULES_CAMS	105	1.06E-02
BIOCARTA_BARRESTIN_SRC_PATHWAY	13	1.06E-02
GO_RESPONSE_TO_FUNGUS	33	1.07E-02
GO_ORGANIC_ACID_BIOSYNTHETIC_PROCESS	208	1.10E-02
GO_NEGATIVE_REGULATION_OF_CELL_PROLIFERATION	480	1.10E-02
GO_REGULATION_OF_SENSORY_PERCEPTION	20	1.12E-02
GO_NEGATIVE_REGULATION_OF_PROTEIN_SECRETION	78	1.13E-02
GO_REGULATION_OF_PH	65	1.14E-02
REACTOME_SPRY_REGULATION_OF_FGF_SIGNALING	13	1.14E-02
GO_VASCULAR_ENDOTHELIAL_GROWTH_FACTOR_RECEPTOR_SIGNALING_PATHWAY	66	1.14E-02
GO_NEGATIVE_REGULATION_OF_TYPE_2_IMMUNE_RESPONSE	8	1.14E-02
REACTOME_CTNNB1_PHOSPHORYLATION_CASCADE	15	1.15E-02
GO_POSITIVE_REGULATION_OF_VIRAL_PROCESS	82	1.18E-02
GO_REGULATION_OF_INTERLEUKIN_1_PRODUCTION	51	1.18E-02
GO_COATED_MEMBRANE	79	1.19E-02
GO_MAP_KINASE_PHOSPHATASE_ACTIVITY	12	1.19E-02
GO_POSITIVE_REGULATION_OF_MESONEPHROS_DEVELOPMENT	10	1.21E-02
GO_REGULATION_OF_REACTIVE_OXYGEN_SPECIES_BIOSYNTHETIC_PROCESS	51	1.21E-02
GO_POSITIVE_REGULATION_OF_CELL_CELL_ADHESION	202	1.23E-02
GO_INTRAMOLECULAR_OXIDOREDUCTASE_ACTIVITY	47	1.24E-02
GO_POSITIVE_REGULATION_OF_EXTRINSIC_APOPTOTIC_SIGNALING_PATHWAY_IN_ABSENCE_OF_LIGAND	13	1.24E-02
GO_MONOSACCHARIDE_BIOSYNTHETIC_PROCESS	46	1.25E-02
KEGG_GLYCOSAMINOGLYCAN_DEGRADATION	19	1.25E-02
GO_POSITIVE_REGULATION_OF_INTRACELLULAR_PROTEIN_TRANSPORT	210	1.27E-02
GO_POSITIVE_REGULATION_OF_EXTRINSIC_APOPTOTIC_SIGNALING_PATHWAY	44	1.28E-02
GO_CELLULAR_RESPONSE_TO_HYDROGEN_PEROXIDE	58	1.28E-02
REACTOME_ASSOCIATION_OF_TRIC_CCT_WITH_TARGET_PROTEINS_DURING_BIOSYNTHESIS	26	1.28E-02
GO_GLUTATHIONE_PEROXIDASE_ACTIVITY	13	1.28E-02
REACTOME_NFKB_AND_MAP_KINASES_ACTIVATION_MEDIATED_BY_TLR4_SIGNALING_REPERTOIRE	68	1.30E-02
GO_REGULATION_OF_CELLULAR_PH	56	1.30E-02
PID_IL12_2PATHWAY	60	1.33E-02
GO_POSITIVE_REGULATION_OF_VASCULATURE_DEVELOPMENT	96	1.34E-02
GO_GLUCOSE_CATABOLIC_PROCESS	23	1.34E-02
GO_POSITIVE_REGULATION_OF_CYTOPLASMIC_TRANSPORT	227	1.35E-02
GO_GRANULOCYTE_ACTIVATION	18	1.39E-02

GO_REGULATION_OF_ESTABLISHMENT_OF_PROTEIN_LOCALIZATION_TO_CHROMOSOME	10	1.39E-02
GO_GOLGI_CISTERNA	82	1.40E-02
GO_CELLULAR_RESPONSE_TO_ABIOTIC_STIMULUS	197	1.40E-02
GO_NEGATIVE_REGULATION_OF_RESPONSE_TO_EXTERNAL_STIMULUS	194	1.41E-02
REACTOME_GLUCONEOGENESIS	26	1.41E-02
GO_NEGATIVE_REGULATION_OF_CELL_GROWTH	125	1.43E-02
GO_POSITIVE_REGULATION_OF_LEUKOCYTE_PROLIFERATION	115	1.43E-02
GO_REGULATION_OF_ALPHA_BETA_T_CELL_ACTIVATION	63	1.45E-02
REACTOME_G1_PHASE	35	1.45E-02
GO_FORMATION_OF_PRIMARY_GERM_LAYER	76	1.45E-02
GO_MACROAUTOPHAGY	220	1.45E-02
GO_REGULATION_OF_TYPE_I_INTERFERON_PRODUCTION	107	1.48E-02
REACTOME_CLASS_A1_RHODOPSIN_LIKE_RECEPTORS	151	1.48E-02
BIOCARTA_HSP27_PATHWAY	14	1.48E-02
GO_KERATAN_SULFATE_BIOSYNTHETIC_PROCESS	16	1.51E-02
GO_REGULATION_OF_DEFENSE_RESPONSE_TO_VIRUS_BY_HOST	101	1.51E-02
GO_UBIQUITIN_LIKE_PROTEIN_LIGASE_BINDING	244	1.54E-02
REACTOME_TRIF_MEDIATED_TLR3_SIGNALING	70	1.54E-02
PID_S1P_S1P2_PATHWAY	24	1.54E-02
GO_PYRIMIDINE_NUCLEOSIDE_METABOLIC_PROCESS	41	1.54E-02
GO_ACTIN_FILAMENT_BUNDLE_ORGANIZATION	42	1.55E-02
GO_REGULATION_OF_PROTEIN_STABILITY	192	1.57E-02
GO_CELLULAR_RESPONSE_TO_MECHANICAL_STIMULUS	61	1.59E-02
GO_REGULATION_OF_DEFENSE_RESPONSE_TO_VIRUS_BY_VIRUS	29	1.62E-02
GO_RESPONSE_TO_PURINE_CONTAINING_COMPOUND	116	1.62E-02
GO_TRANSPORT_VESICLE_MEMBRANE	116	1.62E-02
REACTOME_SIGNALING_BY_EGFR_IN_CANCER	98	1.66E-02
GO_POSITIVE_REGULATION_OF_HOMEOSTATIC_PROCESS	163	1.67E-02
GO_REGULATION_OF_HUMORAL_IMMUNE_RESPONSE	38	1.68E-02
GO_MONOCARBOXYLIC_ACID_CATABOLIC_PROCESS	78	1.69E-02
GO_MULTICELLULAR_ORGANISMAL_HOMEOSTASIS	185	1.69E-02
GO_POSITIVE_REGULATION_OF_INTERLEUKIN_6_PRODUCTION	55	1.69E-02
GO_CLATHRIN_COATED_ENDOCYTIC_VESICLE	49	1.70E-02
GO_WNT_SIGNALING_PATHWAY	273	1.70E-02
GO_POSITIVE_REGULATION_OF_SMOOTH_MUSCLE_CELL_PROLIFERATION	51	1.70E-02
GO_GDP_DISSOCIATION_INHIBITOR_ACTIVITY	10	1.71E-02
GO_PROTEIN_POLYUBIQUITINATION	237	1.71E-02
GO_POSITIVE_REGULATION_OF_MONOCYTE_CHEMOTAXIS	12	1.71E-02
GO_LYMPHOCYTE_MEDIATED_IMMUNITY	114	1.72E-02
GO_REGULATION_OF_PRODUCTION_OF_MOLECULAR_MEDIATOR_OF_IMMUNE_RESPONSE	87	1.73E-02
GO_MYELOID_LEUKOCYTE_MIGRATION	75	1.74E-02
GO_CELLULAR_CARBOHYDRATE_BIOSYNTHETIC_PROCESS	41	1.75E-02
GO_REGULATION_OF_PROTEIN_LOCALIZATION_TO_CHROMOSOME_TELOMERIC_REGION	14	1.75E-02
BIOCARTA_KREB_PATHWAY	8	1.76E-02
GO_FIBROBLAST_GROWTH_FACTOR_RECEPTOR_SIGNALING_PATHWAY	57	1.76E-02
REACTOME_SOS_MEDIATED_SIGNALLING	14	1.76E-02
GO_GOLGI_ASSOCIATED_VESICLE_MEMBRANE	42	1.76E-02
SIG_INSULIN_RECEPTOR_PATHWAY_IN_CARDIAC_MYOCYTES	47	1.76E-02
GO_CELL_REDOX_HOMEOSTASIS	59	1.76E-02
REACTOME_SHC_MEDIATED_SIGNALLING	14	1.76E-02
GO_MODIFICATION_OF_MORPHOLOGY_OR_PHYSIOLOGY_OF_OTHER_ORGANISM	82	1.76E-02
REACTOME_SPHINGOLIPID_METABOLISM	57	1.76E-02
KEGG_GLYCOSPHINGOLIPID_BIOSYNTHESIS_LACTO_AND_NEOLACTO_SERIES	14	1.78E-02
GO_REGULATION_OF_ACTIN_FILAMENT_LENGTH	132	1.78E-02
GO_NEGATIVE_REGULATION_OF_I_KAPPAB_KINASE_NF_KAPPAB_SIGNALING	46	1.78E-02
GO_COGNITION	158	1.79E-02
GO_REGULATION_OF_ACTIN_FILAMENT_BASED_PROCESS	244	1.81E-02
GO_PROTEIN_KINASE_C_BINDING	45	1.82E-02
NABA_SECRETED_FACTORS	163	1.82E-02
GO_LYMPHOCYTE_ACTIVATION	296	1.83E-02
GO_REGULATION_OF_CELL_CELL_ADHESION	314	1.84E-02
GO_SERINE_HYDROLASE_ACTIVITY	130	1.85E-02
BIOCARTA_IL6_PATHWAY	22	1.85E-02
REACTOME_ENDOSOMAL_VACUOLAR_PATHWAY	9	1.86E-02
GO_NEGATIVE_REGULATION_OF_INFLAMMATORY_RESPONSE	76	1.90E-02
GO_MICROVILLUS	51	1.93E-02
REACTOME_TRANS_GOLGI_NETWORK_VESICLE_BUDDING	55	1.94E-02
GO_LEARNING	78	1.94E-02

GO_REGULATION_OF_OSSIFICATION	127	1.94E-02
GO_TRANSLATION_FACTOR_ACTIVITY_RNA_BINDING	82	1.94E-02
GO_NUCLEAR_ENVELOPE_REASSEMBLY	17	1.94E-02
REACTOME_PROCESSING_OF_CAPPED_INTRON_CONTAINING_PRE_MRNA	134	1.95E-02
GO_REGULATION_OF_AUTOPHAGY	231	1.95E-02
REACTOME_SIGNALING_BY_ILS	101	1.99E-02
GO_SMALL_NUCLEAR_RIBONUCLEOPROTEIN_COMPLEX	58	2.00E-02
GO_EXTRACELLULAR_MATRIX	216	2.00E-02
BIOCARTA_NGF_PATHWAY	17	2.02E-02
GO_REGULATION_OF_NITRIC_OXIDE_BIOSYNTHETIC_PROCESS	40	2.07E-02
GO_NEGATIVE_REGULATION_OF_GROWTH	175	2.07E-02
GO_OSSIFICATION	174	2.08E-02
REACTOME_DNA_REPLICATION	179	2.08E-02
GO_POSITIVE_REGULATION_OF_LEUKOCYTE_DIFFERENTIATION	112	2.11E-02
REACTOME_MITOTIC_M_M_G1_PHASES	159	2.12E-02
GO_TUMOR_NECROSIS_FACTOR_RECEPTOR_SUPERFAMILY_BINDING	42	2.14E-02
GO_REGULATION_OF_NOTCH_SIGNALING_PATHWAY	50	2.16E-02
GO_REGULATION_OF_SUPEROXIDE_METABOLIC_PROCESS	17	2.17E-02
GO_MYELOID_LEUKOCYTE_MEDIATED_IMMUNITY	38	2.20E-02
GO_ACTIN_FILAMENT_BASED_PROCESS	358	2.22E-02
GO_SECRETORY_VESICLE	315	2.22E-02
GO_TISSUE_HOMEOSTASIS	120	2.23E-02
KEGG_GLYOXYLATE_AND_DICARBOXYLATE_METABOLISM	14	2.23E-02
GO_MEMBRANE_BUDDING	104	2.28E-02
GO_POSITIVE_REGULATION_OF_SEQUENCE_SPECIFIC_DNA_BINDING_TRANSCRIPTION_FACTOR_ACTIVITY	187	2.29E-02
GO_ER_NUCLEUS_SIGNALING_PATHWAY	31	2.30E-02
HALLMARK_REACTIVE_OXIGEN_SPECIES_PATHWAY	47	2.31E-02
GO_SINGLE_ORGANISM_CELL_ADHESION	357	2.32E-02
REACTOME_CELL_CYCLE_MITOTIC	296	2.32E-02
GO_REGULATION_OF_NF_KAPPAB_IMPORT_INTO_NUCLEUS	42	2.32E-02
GO_REGULATION_OF_PROTEIN_TARGETING	254	2.34E-02
BIOCARTA_CHREBP2_PATHWAY	36	2.35E-02
GO_POSITIVE_REGULATION_OF_CYTOKINE_PRODUCTION_INVOLVED_IN_IMMUNE_RESPONSE	30	2.36E-02
KEGG_PYRUVATE_METABOLISM	33	2.36E-02
GO_MATURATION_OF_SSU_RRNA_FROM_TRICISTRONIC_RRNA_TRANSCRIPT_SSU_RRNA_5_8S_RRNA_LSU_RRNA_	33	2.36E-02
GO_POSITIVE_REGULATION_OF_ALPHA_BETA_T_CELL_ACTIVATION	48	2.36E-02
GO_CHAPERONE_BINDING	73	2.37E-02
REACTOME_RESPONSE_TO_ELEVATED_PLATELET_CYTOSOLIC_CA2_	70	2.38E-02
GO_POSITIVE_REGULATION_OF_CYTOKINE_SECRETION	76	2.38E-02
GO_POSITIVE_REGULATION_OF_SECRETION	258	2.40E-02
PID_PS1_PATHWAY	42	2.40E-02
KEGG_NEUROTROPHIN_SIGNALING_PATHWAY	113	2.40E-02
REACTOME_KERATAN_SULFATE_BIOSYNTHESIS	14	2.40E-02
GO_CELLULAR_RESPONSE_TO_ORGANIC_CYCLIC_COMPOUND	348	2.40E-02
GO_U4_U6_X_U5_TRI_SNRNP_COMPLEX	21	2.41E-02
PID_INTEGRIN_A4B1_PATHWAY	30	2.41E-02
GO_AGING	204	2.43E-02
GO_REGULATION_OF_PEPTIDE_TRANSPORT	182	2.43E-02
REACTOME_GOLGI_ASSOCIATED_VESICLE_BIOGENESIS	48	2.43E-02
GO_NEGATIVE_REGULATION_OF_RESPONSE_TO_ENDOPLASMIC_RETICULUM_STRESS	35	2.44E-02
GO_REGULATION_OF_PODOSOME_ASSEMBLY	12	2.44E-02
GO_CDP_DIACYLGLYCEROL_METABOLIC_PROCESS	14	2.45E-02
GO_REGULATION_OF_VIRAL_TRANSCRIPTION	54	2.46E-02
GO_REGULATION_OF_INTRACELLULAR_PROTEIN_TRANSPORT	319	2.47E-02
PID_RAC1_PATHWAY	52	2.48E-02
KEGG_PENTOSE_AND_GLUCURONATE_INTERCONVERSIONS	9	2.48E-02
GO_WW_DOMAIN_BINDING	23	2.48E-02
GO_REGULATION_OF_TYPE_2_IMMUNE_RESPONSE	22	2.49E-02
GO_MEMBRANE_RAFT_ORGANIZATION	13	2.49E-02
GO_CERAMIDE_CATABOLIC_PROCESS	9	2.50E-02
BIOCARTA_MAPK_PATHWAY	85	2.50E-02
GO_TUMOR_NECROSIS_FACTOR_RECEPTOR_BINDING	28	2.51E-02
REACTOME_INNATE_IMMUNE_SYSTEM	203	2.51E-02
GO_DEFINITIVE_HEMOPOIESIS	16	2.51E-02
REACTOME_PLATELET_ACTIVATION_SIGNALING_AND_AGGREGATION	171	2.51E-02
GO_POSITIVE_REGULATION_OF_IMMUNE_EFFECTOR_PROCESS	137	2.51E-02
GO_PROTEIN_IMPORT_INTO_NUCLEUS_TRANSLOCATION	23	2.52E-02
GO_CELL_CELL_RECOGNITION	36	2.52E-02

REACTOME_MRNA_PROCESSING	152	2.54E-02
GO_POSITIVE_REGULATION_OF_REACTIVE_OXYGEN_SPECIES_METABOLIC_PROCESS	71	2.55E-02
GO_RESPONSE_TO_ALCOHOL	255	2.55E-02
REACTOME_DCC_MEDIATED_ATTRACTIVE_SIGNALING	12	2.55E-02
GO_REGULATION_OF_ESTABLISHMENT_OF_PROTEIN_LOCALIZATION_TO_MITOCHONDRION	114	2.60E-02
REACTOME_TRAF6_MEDIATED_INDUCTION_OF_NFKB_AND_MAP_KINASES_UPON_TLR7_8_OR_9_ACTIVATION	72	2.60E-02
GO_SMALL_GTPASE_MEDIATED_SIGNAL_TRANSDUCTION	305	2.61E-02
GO_MULTI_MULTICELLULAR_ORGANISM_PROCESS	141	2.62E-02
KEGG_CYTOSOLIC_DNA_SENSING_PATHWAY	39	2.65E-02
GO_REGULATION_OF_REACTIVE_OXYGEN_SPECIES_METABOLIC_PROCESS	122	2.65E-02
GO_POSITIVE_REGULATION_OF_CELLULAR_COMPONENT_BIOGENESIS	311	2.65E-02
KEGG_CITRATE_CYCLE_TCA_CYCLE	27	2.68E-02
GO_ROUGH_ENDOPLASMIC_RETICULUM	53	2.69E-02
PID_NCADHERIN_PATHWAY	29	2.69E-02
GO_NEGATIVE_REGULATION_OF_MAPK_CASCADE	120	2.70E-02
GO_REGULATION_OF_PROTEIN_COMPLEX_ASSEMBLY	317	2.70E-02
REACTOME_EARLY_PHASE_OF_HIV_LIFE_CYCLE	14	2.70E-02
REACTOME_MITOCHONDRIAL_FATTY_ACID_BETA_OXIDATION	13	2.70E-02
GO_REGULATION_OF_MONOCYTE_CHEMOTAXIS	15	2.75E-02
GO_CELLULAR_METABOLIC_COMPOUND_SALVAGE	33	2.76E-02
GO_POSITIVE_REGULATION_OF_PHAGOCYTOSIS	39	2.77E-02
GO_REGULATION_OF_SECRETION	481	2.78E-02
GO_GANGLIOSIDE_METABOLIC_PROCESS	19	2.81E-02
GO_MATURATION_OF_SSU_RRNA	41	2.81E-02
GO_NEGATIVE_REGULATION_OF_CALCIUM_MEDIATED_SIGNALING	15	2.83E-02
GO_DNA_DIRECTED_RNA_POLYMERASE_II_CORE_COMPLEX	16	2.83E-02
BIOCARTA_EPHA4_PATHWAY	10	2.84E-02
BIOCARTA_ACTINY_PATHWAY	19	2.84E-02
GO_CELLULAR_MONOVALENT_INORGANIC_CATION_HOMEOSTASIS	71	2.84E-02
GO_TOLL_LIKE_RECEPTOR_SIGNALING_PATHWAY	80	2.85E-02
GO_NEGATIVE_REGULATION_OF_PHOSPHORUS_METABOLIC_PROCESS	416	2.85E-02
GO_XENOPHAGY	77	2.89E-02
NABA_CORE_MATRISOME	139	2.90E-02
GO_REGULATION_OF_MESONEPHROS_DEVELOPMENT	12	2.93E-02
GO_REGULATION_OF_INTERLEUKIN_8_PRODUCTION	51	2.93E-02
KEGG_DRUG_METABOLISM_CYTOCHROME_P450	22	2.93E-02
GO_INTRAMOLECULAR_OXIDOREDUCTASE_ACTIVITY_TRANSPOSING_S_S_BONDS	20	2.96E-02
GO_RESPONSE_TO_KETONE	138	2.97E-02
GO_CLATHRIN_MEDIATED_ENDOCYTOSIS	32	2.98E-02
GO_ACTIN_FILAMENT_ORGANIZATION	144	2.99E-02
GO_DEOXYRIBONUCLEOSIDE_TRIPHOSPHATE_METABOLIC_PROCESS	16	3.00E-02
GO_PEPTIDASE_ACTIVITY	431	3.00E-02
GO_POSITIVE_REGULATION_OF_ERBB_SIGNALING_PATHWAY	28	3.00E-02
PID_GMCSF_PATHWAY	35	3.02E-02
GO_POSITIVE_REGULATION_OF_I_KAPPAB_KINASE_NF_KAPPAB_SIGNALING	164	3.09E-02
GO_REGULATION_OF_EXTRINSIC_APOPTOTIC_SIGNALING_PATHWAY	122	3.12E-02
GO_NEGATIVE_REGULATION_OF_PROTEIN_SERINE_THREONINE_KINASE_ACTIVITY	108	3.12E-02
GO_CELL_KILLING	38	3.12E-02
GO_ANCHORED_COMPONENT_OF_MEMBRANE	80	3.12E-02
GO_MULTIVESICULAR_BODY	29	3.12E-02
GO_NUCLEOTIDE_EXCISION_REPAIR_DNA_DAMAGE_RECOGNITION	23	3.12E-02
GO_RESPONSE_TO_MUSCLE_STRETCH	14	3.16E-02
GO_PEPTIDASE_REGULATOR_ACTIVITY	123	3.17E-02
GO_NUCLEOID	38	3.19E-02
HALLMARK_KRAS_SIGNALING_UP	155	3.23E-02
GO_NEGATIVE_REGULATION_OF_VIRAL_ENTRY_INTO_HOST_CELL	14	3.24E-02
GO_RESPONSE_TO_METAL_ION	238	3.25E-02
GO_ESCRT_III_COMPLEX	10	3.27E-02
GO_TRANSFERASE_ACTIVITY_TRANSFERRING_HEXOSYL_GROUPS	139	3.33E-02
BIOCARTA_FMLP_PATHWAY	32	3.35E-02
REACTOME_LYSOSOME_VESICLE_BIOGENESIS	22	3.35E-02
GO_SMALL_NUCLEOLAR_RIBONUCLEOPROTEIN_COMPLEX	19	3.35E-02
GO_PROTEIN_PHOSPHORYLATED_AMINO_ACID_BINDING	21	3.35E-02
GO_POSITIVE_REGULATION_OF_ALPHA_BETA_T_CELL_DIFFERENTIATION	34	3.35E-02
PID_VEGFR1_2_PATHWAY	65	3.35E-02
BIOCARTA_IL2_PATHWAY	21	3.36E-02
BIOCARTA_CDMAC_PATHWAY	16	3.36E-02
BIOCARTA_CDC42RAC_PATHWAY	16	3.38E-02

BIOCARTA_MAL_PATHWAY	18	3.40E-02
REACTOME_INTEGRATION_OF_PROVIRUS	9	3.41E-02
BIOCARTA_INSULIN_PATHWAY	21	3.41E-02
GO_RNA_SPLICING_VIA_TRANSESTERIFICATION_REACTIONS	251	3.42E-02
PID_INTEGRIN_A9B1_PATHWAY	19	3.42E-02
GO_LIPID_OXIDATION	63	3.44E-02
GO_IMMUNE_SYSTEM_DEVELOPMENT	488	3.44E-02
GO_RESPONSE_TO_LITHIUM_ION	17	3.45E-02
REACTOME_SIGNAL_ATTENUATION	11	3.48E-02
GO_NEGATIVE_REGULATION_OF_INTERLEUKIN_17_PRODUCTION	9	3.48E-02
GO_PROTEIN_TRANSMEMBRANE_TRANSPORT	47	3.49E-02
REACTOME_THE_ROLE_OF_NEF_IN_HIV1_REPLICATION_AND_DISEASE_PATHOGENESIS	28	3.49E-02
GO_REGULATION_OF_COAGULATION	58	3.49E-02
GO_EMBRYO_IMPLANTATION	28	3.53E-02
GO_SPLICEOSOMAL_COMPLEX	163	3.57E-02
GO_RESPONSE_TO_ORGANOPHOSPHORUS	101	3.59E-02
GO_CCR_CHEMOKINE_RECEPTOR_BINDING	21	3.59E-02
GO_BLOOD_MICROPARTICLE	92	3.66E-02
GO_PEPTIDYL_ASPARAGINE_MODIFICATION	34	3.66E-02
HALLMARK_CHOLESTEROL_HOMEOSTASIS	67	3.72E-02
GO_ENZYME_INHIBITOR_ACTIVITY	240	3.73E-02
GO_ATPASE_ACTIVITY_COUPLED_TO_MOVEMENT_OF_SUBSTANCES	96	3.76E-02
GO_MONOVALENT_INORGANIC_CATION_HOMEOSTASIS	85	3.76E-02
GO_OPSONIN_BINDING	8	3.77E-02
GO_MACROMOLECULE_TRANSMEMBRANE_TRANSPORTER_ACTIVITY	19	3.79E-02
GO_POSITIVE_REGULATION_OF_INTERFERON_GAMMA_PRODUCTION	57	3.80E-02
REACTOME_DEVELOPMENTAL_BIOLOGY	286	3.80E-02
GO_POSITIVE_REGULATION_OF_ENDOCYTOSIS	89	3.82E-02
GO_REGULATION_OF_ALPHA_BETA_T_CELL_DIFFERENTIATION	41	3.82E-02
GO_RESPONSE_TO_STEROID_HORMONE	353	3.83E-02
HALLMARK_MYC_TARGETS_V2	58	3.83E-02
REACTOME_SIGNALLING_TO_ERKS	32	3.84E-02
GO_ADP_METABOLIC_PROCESS	37	3.84E-02
REACTOME_CELL_CYCLE	374	3.87E-02
GO_MEMBRANE_INVAGINATION	40	3.87E-02
GO_CELLULAR_DEFENSE_RESPONSE	48	3.87E-02
GO_PYRIMIDINE_NUCLEOSIDE_BIOSYNTHETIC_PROCESS	26	3.88E-02
GO_REGULATION_OF_B_CELL_ACTIVATION	105	3.88E-02
PID_PI3KCI_AKT_PATHWAY	35	3.88E-02
GO_FATTY_ACID_CATABOLIC_PROCESS	67	3.90E-02
KEGG_FC_GAMMA_R_MEDIATED_PHAGOCYTOSIS	93	3.90E-02
BIOCARTA_MET_PATHWAY	36	3.90E-02
GO_POSITIVE_REGULATION_OF_B_CELL_ACTIVATION	72	3.92E-02
GO_NOTCH_SIGNALING_PATHWAY	82	3.93E-02
BIOCARTA_BIOPEPTIDES_PATHWAY	36	3.94E-02
GO_ESCRT_COMPLEX	23	3.94E-02
REACTOME_P75NTR_SIGNALS_VIA_NFKB	12	3.95E-02
GO_CHEMOKINE_RECEPTOR_BINDING	39	3.95E-02
GO_INFLAMMASOME_COMPLEX	11	3.97E-02
GO_SOMATIC_STEM_CELL_POPULATION_MAINTENANCE	47	3.97E-02
GO_NUCLEOBASE_CONTAINING_SMALL_MOLECULE_INTERCONVERSION	20	3.98E-02
GO_CELL_BODY	343	3.99E-02
GO_TRICARBOXYLIC_ACID_METABOLIC_PROCESS	32	3.99E-02
GO_ENDOPEPTIDASE_ACTIVITY	280	3.99E-02
GO_REGULATION_OF_CHEMOTAXIS	133	4.00E-02
GO_RETROGRADE_VESICLE_MEDIATED_TRANSPORT_GOLGI_TO_ER	69	4.00E-02
GO_NEGATIVE_REGULATION_OF_PEPTIDASE_ACTIVITY	149	4.00E-02
GO_ENDOPLASMIC_RETICULUM_GOLGI_INTERMEDIATE_COMPARTMENT	90	4.01E-02
GO_POSITIVE_REGULATION_OF_INTERLEUKIN_6_SECRETION	11	4.01E-02
REACTOME_TOLL_RECEPTOR_CASCADES	111	4.01E-02
GO_CELLULAR_RESPONSE_TO_LIPOPROTEIN_PARTICLE_STIMULUS	11	4.05E-02
GO_GABAERGIC_NEURON_DIFFERENTIATION	6	4.05E-02
REACTOME_CHEMOKINE_RECEPTORS_BIND_CHEMOKINES	40	4.05E-02
BIOCARTA_SPRY_PATHWAY	15	4.05E-02
GO_CELLULAR_ALDEHYDE_METABOLIC_PROCESS	67	4.05E-02
GO_LIPID_CATABOLIC_PROCESS	172	4.07E-02
REACTOME_NEF_MEDIATES_DOWN_MODULATION_OF_CELL_SURFACE_RECEPTORS_BY_RECRUITING_THEM_TO_CLATHRIN_ADAPTERS	21	4.08E-02
GO_MULTIVESICULAR_BODY_ORGANIZATION	29	4.11E-02

REACTOME_CELL_JUNCTION_ORGANIZATION	43	4.11E-02
GO_NEGATIVE_REGULATION_OF_NEURON_DEATH	129	4.11E-02
GO_CELL_CELL_ADHESION	400	4.14E-02
GO_COMPLEMENT_BINDING	15	4.23E-02
GO_ADAPTIVE_IMMUNE_RESPONSE_BASED_ON_SOMATIC_RECOMBINATION_OF_IMMUNE_RECEPTORS_BUILT_FROM_IMMUNOGLOBULIN_SUPERFAMILY_DOI	126	4.23E-02
REACTOME_SHC1_EVENTS_IN_ERBB4_SIGNALING	17	4.23E-02
GO_NUCLEOSOMAL_DNA_BINDING	28	4.24E-02
GO_FIBRIL_ORGANIZATION	15	4.24E-02
GO_EXOPEPTIDASE_ACTIVITY	75	4.24E-02
GO_MEMBRANE_LIPID_METABOLIC_PROCESS	150	4.26E-02
GO_CELLULAR_RESPONSE_TO_REACTIVE_OXYGEN_SPECIES	92	4.26E-02
GO_POSITIVE_REGULATION_OF_SPROUTING_ANGIOGENESIS	11	4.27E-02
BIOCARTA_ETC_PATHWAY	11	4.28E-02
GO_PHAGOCYTOSIS_ENGULFMENT	30	4.29E-02
BIOCARTA_FEEDER_PATHWAY	7	4.29E-02
GO_HYDROGEN_PEROXIDE_METABOLIC_PROCESS	21	4.29E-02
KEGG_CYTOKINE_CYTOKINE_RECEPTOR_INTERACTION	178	4.32E-02
REACTOME_ACTIVATED_TLR4_SIGNALLING	88	4.34E-02
GO_PYRIMIDINE_CONTAINING_COMPOUND_BIOSYNTHETIC_PROCESS	35	4.38E-02
GO_REGULATION_OF_INTERLEUKIN_1_BETA_PRODUCTION	43	4.38E-02
SA_MMP_CYTOKINE_CONNECTION	15	4.39E-02
GO_EPHRIN_RECEPTOR_BINDING	19	4.40E-02
GO_POSITIVE_REGULATION_OF_MESENCHYMAL_CELL_PROLIFERATION	16	4.40E-02
REACTOME_SYNTHESIS_OF_SUBSTRATES_IN_N_GLYCAN_BIOSYTHESIS	13	4.41E-02
GO_ORGANOPHOSPHATE_BIOSYNTHETIC_PROCESS	373	4.43E-02
KEGG_PROPANOATE_METABOLISM	30	4.44E-02
GO_NEGATIVE_REGULATION_OF_NOTCH_SIGNALING_PATHWAY	18	4.44E-02
GO_REGULATION_OF_NEURON_DEATH	197	4.47E-02
PID_ENDOTHELIN_PATHWAY	53	4.48E-02
GO_RESPONSE_TO_CARBOHYDRATE	119	4.49E-02
GO_ANGIOGENESIS_INVOLVED_IN_WOUND_HEALING	9	4.51E-02
GO_CIS_TRANS_ISOMERASE_ACTIVITY	36	4.52E-02
GO_INSULIN_LIKE_GROWTH_FACTOR_RECEPTOR_BINDING	10	4.53E-02
KEGG_REGULATION_OF_ACTIN_CYTOSKELETON	168	4.58E-02
GO_NEGATIVE_REGULATION_OF_PROTEOLYSIS	219	4.59E-02
GO_CELLULAR_RESPONSE_TO_KETONE	60	4.60E-02
HALLMARK_XENOBIOTIC_METABOLISM	159	4.71E-02
GO_NEGATIVE_REGULATION_OF_REACTIVE_OXYGEN_SPECIES_BIOSYNTHETIC_PROCESS	9	4.75E-02
GO_PROTEOGLYCAN_BIOSYNTHETIC_PROCESS	38	4.75E-02
REACTOME_SHC_RELATED_EVENTS	15	4.78E-02
REACTOME_AXON_GUIDANCE	183	4.79E-02
GO_DEFENSE_RESPONSE_TO_VIRUS	130	4.83E-02
GO_RESPONSE_TO_HEAT	66	4.84E-02
GO_RESPONSE_TO_THYROID_HORMONE	18	4.84E-02
PID_SHP2_PATHWAY	47	4.85E-02
PID_NFAT_3PATHWAY	52	4.90E-02
REACTOME_PLATELET_SENSITIZATION_BY_LDL	15	4.91E-02
HALLMARK_PI3K_AKT_MTOR_SIGNALING	95	4.94E-02
REACTOME_HIV_LIFE_CYCLE	111	4.97E-02
REACTOME_SIGNALING_BY_ERBB2	87	4.98E-02
REACTOME_PYRIMIDINE_METABOLISM	20	4.98E-02
PID_CERAMIDE_PATHWAY	46	4.99E-02

Table S3. GSEA list of differentially down-regulated processes (FDR < 0.05) in monocytes of CF subjects after treatment with ivacaftor for 7 days.

Gene Set Name	Number of Genes	FDR
REACTOME_GENERIC_TRANSCRIPTION_PATHWAY	305	0
GO_CHROMATIN_MODIFICATION	475	1.43E-02
GO_CENTRIOLE	92	4.76E-02

Table S4. Functional enrichment analysis of DEGs with increased expression in responders vs. non-responders in the GOAL PBMC transcriptomic analysis (Sun, et al).

Gene Ontology ID	Description	Enrichment Ratio	P-value	FDR
GO:0045055	regulated exocytosis	6.081	3.31E-08	3.01E-04
GO:0006887	exocytosis	5.326	1.71E-07	5.78E-04
GO:0016192	vesicle-mediated transport	3.503	1.91E-07	5.78E-04
GO:0032940	secretion by cell	3.928	4.65E-07	1.06E-03
GO:0043312	neutrophil degranulation	7.056	1.11E-06	1.43E-03
GO:0002283	neutrophil activation involved in immune response	7.013	1.18E-06	1.43E-03
GO:0042119	neutrophil activation	6.885	1.39E-06	1.43E-03
GO:0002446	neutrophil mediated immunity	6.857	1.44E-06	1.43E-03
GO:0036230	granulocyte activation	6.802	1.55E-06	1.43E-03
GO:0046903	secretion	3.603	1.57E-06	1.43E-03
GO:0098657	import into cell	5.153	2.22E-06	1.83E-03
GO:0043299	leukocyte degranulation	6.430	2.57E-06	1.95E-03
GO:0002275	myeloid cell activation involved in immune response	6.310	3.04E-06	2.13E-03
GO:0002444	myeloid leukocyte mediated immunity	6.207	3.52E-06	2.29E-03
GO:0002274	myeloid leukocyte activation	5.365	1.27E-05	7.69E-03
GO:0006897	endocytosis	5.039	2.18E-05	1.24E-02
GO:0002366	leukocyte activation involved in immune response	4.908	2.73E-05	1.45E-02
GO:0002263	cell activation involved in immune response	4.880	2.87E-05	1.45E-02
GO:0002443	leukocyte mediated immunity	4.475	5.98E-05	2.86E-02
GO:0002252	immune effector process	3.577	8.63E-05	3.92E-02
